# M^2^aia—Interactive, fast, and memory-efficient analysis of 2D and 3D multi-modal mass spectrometry imaging data

**DOI:** 10.1093/gigascience/giab049

**Published:** 2021-07-20

**Authors:** Jonas Cordes, Thomas Enzlein, Christian Marsching, Marven Hinze, Sandy Engelhardt, Carsten Hopf, Ivo Wolf

**Affiliations:** Faculty of Computer Science, Mannheim University of Applied Sciences, Paul-Wittsack-Straße 10, 68163 Mannheim, Germany; Medical Faculty Mannheim, University Heidelberg, Theodor Kutzer-Ufer 1-3, 68167 Mannheim, Germany; Center for Mass Spectrometry and Optical Spectroscopy (CeMOS), Mannheim University of Applied Sciences, Paul-Wittsack-Straße 10, 68163 Mannheim, Germany; Center for Mass Spectrometry and Optical Spectroscopy (CeMOS), Mannheim University of Applied Sciences, Paul-Wittsack-Straße 10, 68163 Mannheim, Germany; Faculty of Computer Science, Mannheim University of Applied Sciences, Paul-Wittsack-Straße 10, 68163 Mannheim, Germany; Working Group “Artificial Intelligence in Cardiovascular Medicine” (AICM), University Hospital Heidelberg, Im Neuenheimer Feld 410, 69120 Heidelberg, Germany; Center for Mass Spectrometry and Optical Spectroscopy (CeMOS), Mannheim University of Applied Sciences, Paul-Wittsack-Straße 10, 68163 Mannheim, Germany; Faculty of Computer Science, Mannheim University of Applied Sciences, Paul-Wittsack-Straße 10, 68163 Mannheim, Germany

**Keywords:** mass spectrometry imaging, multi-modal, image registration, image reconstruction, three-dimensional, interactive visualization

## Abstract

**Background:**

Mass spectrometry imaging (MSI) is a label-free analysis method for resolving bio-molecules or pharmaceuticals in the spatial domain. It offers unique perspectives for the examination of entire organs or other tissue specimens. Owing to increasing capabilities of modern MSI devices, the use of 3D and multi-modal MSI becomes feasible in routine applications—resulting in hundreds of gigabytes of data. To fully leverage such MSI acquisitions, interactive tools for 3D image reconstruction, visualization, and analysis are required, which preferably should be open-source to allow scientists to develop custom extensions.

**Findings:**

We introduce M^2^aia (MSI applications for interactive analysis in MITK), a software tool providing interactive and memory-efficient data access and signal processing of multiple large MSI datasets stored in imzML format. M^2^aia extends MITK, a popular open-source tool in medical image processing. Besides the steps of a typical signal processing workflow, M^2^aia offers fast visual interaction, image segmentation, deformable 3D image reconstruction, and multi-modal registration. A unique feature is that fused data with individual mass axes can be visualized in a shared coordinate system. We demonstrate features of M^2^aia by reanalyzing an N-glycan mouse kidney dataset and 3D reconstruction and multi-modal image registration of a lipid and peptide dataset of a mouse brain, which we make publicly available.

**Conclusions:**

To our knowledge, M^2^aia is the first extensible open-source application that enables a fast, user-friendly, and interactive exploration of large datasets. M^2^aia is applicable to a wide range of MSI analysis tasks.

## Introduction

Imaging of molecular information in the spatial domain enables insights into otherwise hidden conditions and metabolic processes. Mass spectrometry imaging (MSI) represents a class of label-free and spot-wise spectrometry acquisition techniques [1] and already plays an important role in a wide range of biomedical and industrial applications. It is a proven technique used for development of pharmacological agents [2] or for phenotyping of pathological tissue samples [3]. The spotwise imaging process results in 2D MSI data, where in each pixel a spectrum is acquired representing the relative intensities of ionizable molecular compounds covering a wide range of mass to charge (*m*/*z*) ratios.

It is expected that the use of MSI techniques will increase dramatically in the future owing to the development of faster acquisition techniques and improved accuracy of MSI devices [4]. The constantly growing file sizes of up to hundreds of gigabytes are a challenge for data processing, especially for interactive tasks like visualization and exploratory data analysis [5].

The research community can choose from a variety of software tools for MSI data [6]. Interactive exploration, analysis, and processing of MSI data cannot be performed by the currently available open-source software solutions because they are not designed for an interactive scenario where low latencies and a user-friendly graphical interface are desirable. On the other hand, interactive and fast feedback is important for successful experiments, allowing early intervention by inclusion of interesting or exclusion of invalid image regions in further analysis. If blackbox-like scripts are just applied, the traceability and quality of MSI data processing steps can deteriorate. Apart from that, exporting data of intermediate steps for visualization in external tools is time consuming, error prone, and difficult to handle, especially if running on a server infrastructure.

The emerging field of 3D MSI [7–10] offers new perspectives into the molecular structure of biological samples. Images created by 3D mass spectrometry (MS) can be generated by combining multiple adjacent 2D MS images of consecutive cuts of a biological sample to an MS image volume by co-registration (3D image reconstruction) [5,11,12].

Multi-modal MSI can refer to (i) imaging of lipids, peptides, and proteins on adjacent tissue sections or the same tissue section [13–16] with intermediate removal of the matrix [17] (to apply different matrix preparation approaches) or (ii) the registration of MSI and another imaging modality like optical imaging [18–21]. Registration of multi-modal data is challenging because of modality-dependent image contrasts. Especially difficult is to eliminate distortions caused by the preparation process, partial destruction in the original tissue morphology, and spatially misplaced tissue sections (rotated or placed upside down). Because it is an emerging field of research, interactive environments for (semi-) automatic 3D reconstruction or multi-modal MSI experiments do not exist yet in an openly accessible and mature form. The currently most advanced commercial solution is SCiLS Lab (Bruker Daltonik GmbH, Bremen, Germany).

To recover the correct spatial relationship between corresponding images, registration methods need to be applied to perform mirroring, rigid or deformable image transformations. Open-source toolkits and command-line applications targeting intensity-based image registration are available, e.g., the Insight Toolkit (ITK) [22,23] and elastix [24], and have been shown to be applicable to multi-modal image registration [19] and 3D MSI image reconstruction [12,25].

The results of 2D/3D MSI produce large hyper-dimensional datasets that pose computational challenges to interactive visualization, exploration, and analysis on commonly available software and hardware configurations. Software solutions for MSI are often limited by available RAM and time-consuming initialization procedures, which is especially true in the case of 3D image reconstruction where preferably all required 2D MSI datasets are accessible at the same time.

### Aims

**Figure 1:**
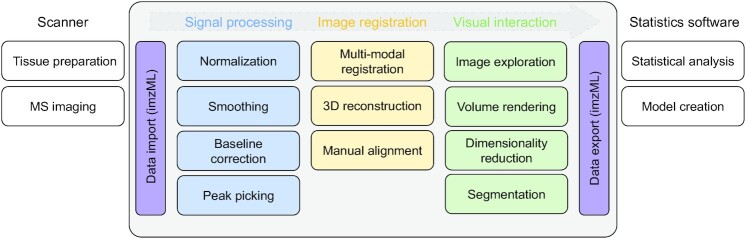
Commonly used MSI pipeline (left to right). The aim of M^2^aia is to provide an interactive environment with support for all image-related tasks from data import to co-registration, exploration, segmentation, and data export.

We created M^2^aia (MSI application for interactive analysis in MITK, RRID:SCR_019324, biotools:m2aia) by integrating MSI support into the platform-independent and open-source Medical Imaging Interaction Toolkit (MITK) [26]. M^2^aia supports the major MS image-related processing tasks required in an MSI study within a single framework. These are illustrated in Fig. 1. M^2^aia supports read/write access to MSI data in the open standard format imaging mz Markup Language (imzML) [27] supporting 2D/3D continuous and processed datasets, in addition to the (medical) image and image-related file formats supported by MITK. We developed M^2^aia with 4 main goals in mind: (i) providing a complete set of memory-efficient and fast MSI utilities for interactive data visualization, signal processing, and analysis, optionally usable for batch-processing; (ii) simultaneous handling of multiple, potentially multi-modal images with minimal memory overhead; (iii) support for user-driven 3D MS image reconstruction and multi-modal image registration; and (iv) the distribution of a community extendable and open-source code base.

In contrast to applications like Galaxy [28] that provide a server-side solution for non-interactive standardized processing of large MSI studies [29], M^2^aia is designed to give a fast, visual, and interactive user response on commonly available hardware offering the aforementioned features. All main features can be accessed by means of a user-friendly graphical interface, and, therefore, no programming skills are required. Thus, the MSI extension of Galaxy [29] and M^2^aia complement each other, offering different tools for a full-stack open-source MSI working environment.

### The Medical Imaging Interaction Toolkit as an application and development backbone

MITK [26] is a popular open-source toolkit for development of interactive medical image processing applications. In essence, the MITK framework is characterized by (i) a flexible plugin-based user-interface and modular system environment; (ii) the capability of handling a wide range of 2D, 3D, and 3D+t (medical) images and image-related data such as surfaces, point sets, and image segmentations; (iii) consistent interactive visualization of multi-planar reconstructions and 3D visualizations; and (iv) a completely open-source code base built on top of ITK [22, 23], VTK [30,31], and Qt [32]. MITK is a cross-platform C++ toolkit and officially supports Windows, Linux, and macOS. A modular software structure allows the development of new applications decoupled from the main source tree of MITK. The Joint Imaging Platform (JIP) [33] provides methods to containerize MITK, allowing it to be hosted on a scalable server infrastructure, and offers Virtual Network Computing as an interface able to stream MITK to a Web browser.

## Findings

**Figure 2:**
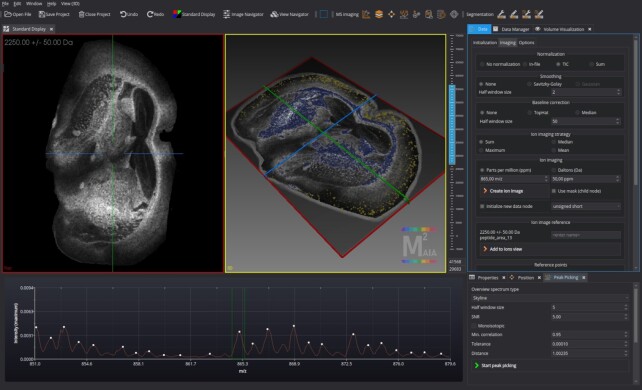
Graphical user interface. M^2^aia introduces utilities for common MSI processing tasks and new workflows for 3D reconstruction and multi-modal image registration, extending the standard features of MITK [16] such as semi-automated segmentation and 2D/3D visualization. Rendered images show a multi-modal 3D reconstructed volume of APP NL-G-F mice brain lipid and peptide MALDI-TOF datasets introduced in this article. Volume rendering was applied to visualize high intensities of *m*/*z* 865 ± 0.4 Da (blue) of the 3D reconstructed lipid dataset and *m*/*z* 4070 ± 5 Da (yellow) of the 3D reconstructed peptide dataset. The plugin for controlling the import and processing of MSI data is shown on the upper right side, the peak picking plugin on the lower right corner, and the interactive spectrum view plugin on the lower left (here displaying the skyline spectrum of a lipid MS image).

M^2^aia can be considered as an interactive open-source exploration and analysis application for MSI data with the capability of 3D image reconstruction and multi-modal image registration that is extensible by the community. Figure 2 gives an impression of the graphical user interface (GUI). In the following sections we introduce the main concepts and newly implemented features that are introduced by M^2^aia. These features were compiled into several plugins targeting different aspects of MSI processing, including plugins for MSI import, export, signal processing, peak picking, multi-modal registration, and 3D reconstruction. All MSI-related plugins were built from scratch (except parts that incorporate third-party technologies) and highly optimized for fast reaction times and low memory overhead. Thanks to the multi-platform paradigm of MITK, binaries for multiple operating systems, including Windows- and Unix-based systems, are available. In addition, the modular system of M^2^aia allows the community a highly flexible development of plugins to add new functionalities and to develop command-line–based applications for batch processing.

Finally, we provide 2 state-of-the-art use-cases. The first use-case demonstrates an *m*/*z* candidate detection task on publicly available N-linked glycan data [34] (available in the PRIDE repository [35]). In the second use-case we elaborate on the 3D reconstruction capabilities of M^2^aia using a multi-modal 3D MSI dataset of lipid and peptide acquisitions, which we make publicly available in the GigaScience respository, GigaDB [36]. Especially, the interactive way of visualizing 2D/3D multi-modal image registration results with individual mass axes in a shared coordinate system is a unique feature, and, to our knowledge, M^2^aia is the first open-source application that offers this kind of visual interaction.

### Data handling

We implement support for the imzML file format (continuous-profile, continuous-centroid, and processed-centroid with external data storage in an imaging binary data [*.ibd] file). Provided that MSI data can be converted into the imzML format, M^2^aia is capable of processing data of various MSI devices, e.g., matrix-assisted laser desorption/ionization (MALDI), desorption electrospray ionization (DESI), or secondary ion mass spectrometry (SIMS).

One challenge in MSI is the handling of computing resources. Loading tens of gigabytes of data into the computer’s memory is almost impossible without server-side processing capabilities. To face this challenge, we follow the strategy of “lazy loading,” resulting in minimal memory overhead and reaction times.

**Figure 3:**
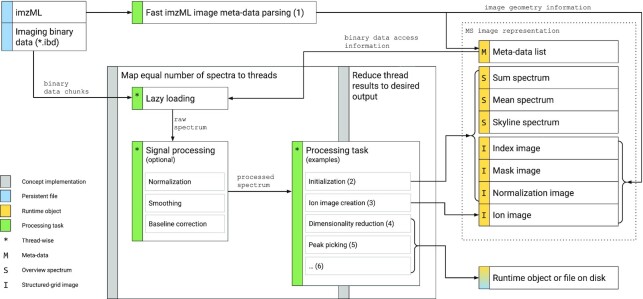
Data flow for processing imzML files in M^2^aia. Fast imzML image metadata parsing (1) provides information about spectra and the image geometry. Spectra processing tasks 2–6 take advantage of the concept to assign equal parts of spectra to different threads. Each thread can trigger loading of spectra from disk (lazy loading). Signal processing can optionally be invoked. Processing task-specific reduction of thread results produces the desired output. Spectrum data are discarded after processing and only the processing results are stored to keep memory consumption low.

Internally in M^2^aia the access to the imzML data is split into 2 steps. First, all necessary metadata *M* are read from the XML file (*.imzML), which contains both the image geometry information and all access information of the spectra (Fig. 3, data processing task 1). Because the imzML files can become big XML structures (especially in 3D MSI several gigabytes), processing these files is very slow with conventional XML parsers. To overcome this problem we implemented an approach based on line-wise parsing, reducing the consumed time to a minimum. Second, an equal number of spectra are assigned to multiple threads, where each can read spectral data on demand (lazy loading) from the imaging binary data (*.ibd) container, using the metadata *M* from the first step (Fig. 3, data processing task marked with an asterisk). From this point on, the processing time depends on the user-defined signal processing and data processing task–related steps. Loaded binary data blocks (e.g., partial spectrum data) are discarded after processing. Only the resulting numbers, e.g., the values of scaling factors, overview spectra, or pixels, are retained.

In addition to the aforementioned metadata content *M*, a set of gray-scale image artefacts $\hat{I}$ and a set of overview spectrum artefacts $\hat{S}$ are kept in memory.

The set of image artefacts $\hat{I}=\lbrace {I^{\mathrm{ion}}}, {I^{\mathrm{norm}}}, {I^{\mathrm{index}}}, {I^{\mathrm{mask}}}\rbrace$ includes *I*^ion^ as placeholder image for representation of ion images (data type: double), *I*^norm^ as placeholder image of spectra-related normalization factors (data type: double), *I*^index^ as an image with pixel positions according to the spectrum ID in the imzML XML structure (data type: int), and *I*^mask^ as a binary mask indicating where valid spectrum information is accessible (data type: short). All images have the same number of pixels |*I*|.

The set of overview spectra $\hat{S}=\lbrace {S^{\mathrm{skyline}}}, {S^{\mathrm{sum}}}, {S^{\mathrm{mean}}}\rbrace$ includes *S*^skyline^ as placeholder for the representation of the maximum spectrum, *S*^sum^ for the sum spectrum, and *S*^mean^ for the mean spectrum over all spectra in the image. All overview spectra have the same spectral depth |*S*| and data type double.

$\hat{I}$ and $\hat{S}$ are generated once during the initialization of an MSI image in M^2^aia and are held in memory as long as the MSI image is loaded. These artefacts require only a fraction of memory compared to loading the whole MSI spectra data. Data items are illustrated in Fig. 3.

The total memory *E* required for imaging and spectral data can be approximated with respect to the number of pixels of a placeholder image |*I*|, spectrum depth |*S*|, and the sizes δ^type^ of the numeric types of the system in bytes. Additionally, during the multi-threaded processing, *T* threads are initialized, each of which at least allocates a single spectrum of size |*S*|:

(1) \begin{eqnarray*}
E = |I|* \left(2\delta ^{\mathrm{double}}+\delta ^{\mathrm{int}} +\delta ^{\mathrm{short}}\right) + (3*|S|+T*|S|)*\delta ^{\mathrm{double}}\mathrm{.}
\end{eqnarray*}

Consequently, the memory required by M^2^aia is *O*(|*I*| + |*S*|) and thus much lower than the total size of binary data, which is *O*(|*I*|*|*S*|).

To test the data handling of M^2^aia, we conducted performance measurements for processing steps 1–3 (see Fig. 3) using the publicly available 3D reference datasets described in [5]. The list of the used reference datasets and the results are reported in Table 1. The experiments were repeated 3 times each to obtain mean processing times and memory usage.

**Table 1.** Timing experiments on mobile and desktop systems using 3D reference data

File name	System	Size (GB)	Spectra	Depth	Parsing of metadata (1) (s)	Initialization (2) (s)	Creation of ion image (3) (s)	RAM usage (MB)
3D Mouse Kidney	A	44.2	1,362,830	7,671	24.9	26.27	10.9	435.1
3D Mouse Kidney	B	44.2	1,362,830	7,671	5.7	2.1	0.6	435.1
3D Mouse Pancreas	A	27.3	497,225	13,297	9.4	13.14	2.8	292.4
3D OSCC	A	26.8	828,558	7,665	14.9	13.4	4.04	323.5
Microbe Interaction 3D Timecourse	A	2.9	17,672	40,299	0.4	0.9	0.08	231.8

Processing of 3D reference datasets published by Oetjen et al. [5] and available in the MetaboLights repository [MTBLS176]. The table lists the file names, number of spectra, depth of a spectrum, and the mean time in seconds and memory usage including memory used by the GUI values of 3 manually repeated runs. Processes 1–3 according to the procedures shown in Fig. 3. Applied signal processing includes total-ion-count (TIC) normalization. System configuration A: mobile PC, Intel® Core^TM^ i7-8750H CPU at 2.20  GHz 6-core processor, 16 GB physical memory, and SSD. System configuration B: desktop PC, AMD® Ryzen 9 5900x CPU at 3.7  GHz 12-core processor, 32 GB physical memory, and M.2 SSD.

### Signal processing methods

Signal processing is a fundamental and important step in MSI because spectral analysis may be influenced by a wide range of factors including sample preparation, acquisition methods, chemical noise, analyte displacement, and inconsistent intensities due to matrix or surface inhomogeneity, electronic fluctuations, or ionization effects [2]. Signal processing aims to reduce these influences. Therefore, M^2^aia offers all steps of a typical signal processing workflow including (i) normalization, (ii) noise reduction, (iii) baseline correction, and (iv) peak-picking methods.

#### Normalization/calibration

To compensate for pixel-to-pixel intensity variations, a spectrum *S* is normalized by

(2) \begin{eqnarray*}
S(j)=S^{\mathrm{old}}(j)*f_{S}^{-1}\mathrm{,}
\end{eqnarray*}

where *S*^old^(*j*) and *S*(*j*) are the original and scaled intensities, respectively; *f* is the normalization value; and *j* is the index related to the *m*/*z* position in a spectrum. If no normalization is applied, *f* is set to 1. Generated normalization maps are accessible for each image. As methods for determining *f_S_*, M^2^aia offers calculating total-ion-count (TIC), the median, or the use of in-file normalization values (defined spectrum-wise in imzML, if available).

#### Noise reduction

Spectrum-wise noise reduction is realized via the Savitzky and Golay filter [37] method.

#### Baseline correction

Most signal-processing stages take advantage of baseline-corrected spectra. In MS, the baseline is the smooth curve offsetting the actual intensities. Baseline correction is generally performed by subtracting the estimated baseline from the intensity spectrum. Implemented is baseline estimation using the top-hat [38] or running median method.

#### Peak picking

Peak picking refers to the detection of peaks in a spectrum and provides information about the *m*/*z*-values and corresponding intensities of the peaks. In M^2^aia, peaks within a spectrum are detected by finding local maxima above a certain noise level using a sliding window approach. The noise level is estimated by the median absolute deviation. Monoisotopic peaks can be identified by automatic Poisson peak harvesting [39].

## Image interaction and processing

Key advantages of M^2^aia are its interactive visualization and image-based processing methods. Visual interaction with 2D and 3D data allows the exploration and perception of whole datasets. Dimensionality reduction methods can be applied to generate views that help to exploit the full potential of MSI. Methods for image segmentation can be used to restrict the analysis to relevant regions of interest. Image- and/or point-based registration methods enable the creation of 3D reconstructions of stacks of MSI images or image registration in multi-modal imaging setups. These concepts are described in more details below.

### Data visualization and interaction concepts

**Figure 4:**
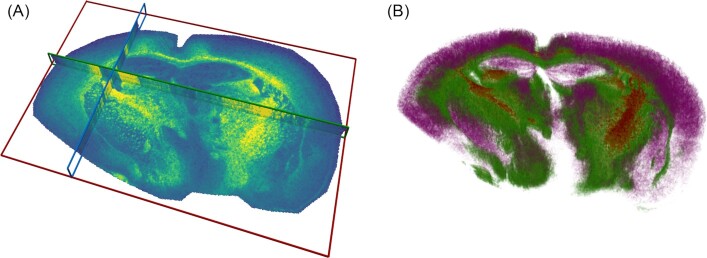
3D reconstruction of lipid MALDI-MS TOF images of 10 consecutive APP NL-G-F mouse brain tissue sections. (A) Multi-planar reconstruction of the 3D MS image showing ion images at *m*/*z* 865.05 ± 0.1 Da. (B) Volume visualization of different mass features. High intensities (green) and low intensities (red) in ion image at *m*/*z* 865.05 ± 0.1 Da are visualized. Additionally, the cortex is highlighted by high intensities at *m*/*z* 868.76 ± 0.1 Da (purple). Processing and visualization have been performed completely within M^2^aia.

All images loaded to M^2^aia are represented in a common virtual world coordinate system. This world space can be observed through multiple render windows that are showing different sliced views of the world space (multi-planar reconstructions, e.g., top views and side views of 3D stacks; tilted views are also possible). Complementary to the planar views, a 3D view is provided, facilitating the perception of the distribution of structures in 3D space. Additional to the visualization of pixel image data, M^2^aia also supports the incorporation of surfaces (e.g., created by segmentation of structures or loaded from files after external processing) and offers volume rendering for 3D image data (Fig. 4). Several color maps are available and can be applied to images individually.

### Image segmentation

Image segmentation can be used for selecting regions or structures within an image. M^2^aia offers semi-automatic segmentation tools for 2D/3D image data. Segmented areas can be statistically analyzed within M^2^aia or exported to obtain insights into the local ion intensity distributions.

### Dimensionality reduction

Various dimensionality reduction (DR) methods are available in M^2^aia for the investigation of the high-dimensional MSI data. The most basic DR method is the extraction of ion images by an intensity transformation within an interactively selected window of *m*/*z* values. The intensity values within the window are transformed into a single representative value. Currently, M^2^aia allows the calculation of mean, maximum, sum, or median for this purpose.

**Figure 5:**
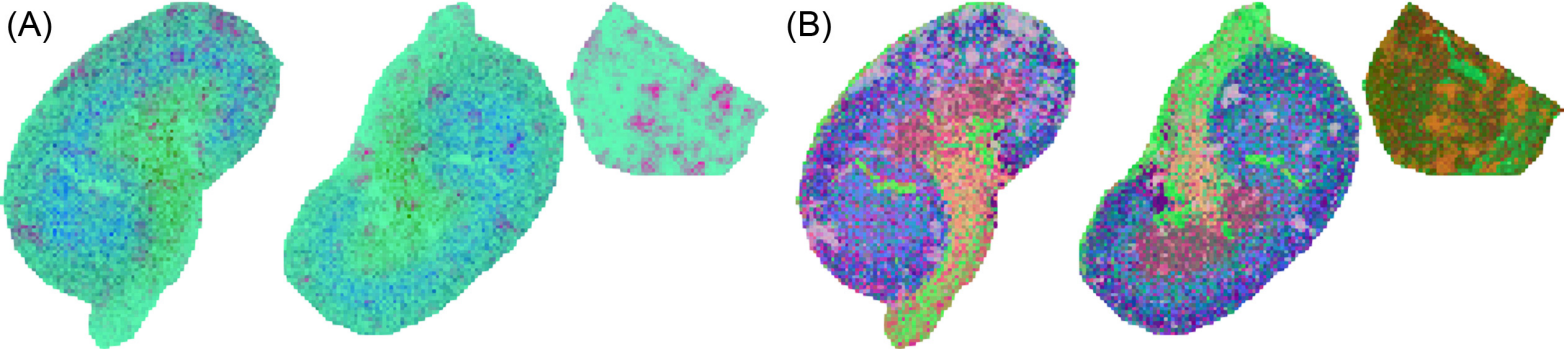
Results of 2 dimensionality reduction methods after performing the pipeline shown in Fig. 6. (A) The 3 principal components with the largest eigenvalues of a PCA. (B) Results of a t-SNE with a target dimension of 3.

More complex DR methods enable capturing features of the dataset distributed over many ion images at once. Principal component analysis (PCA) as a linear and t-stochastic neighbourhood embedding (t-SNE) [40] as a non-linear DR method are available in M^2^aia. For initialization of the PCA or t-SNE, a finite set of ion images must be provided. Figure 5 illustrates the application of PCA and t-SNE to a centroid dataset.

### Image registration

Reconstruction of 3D MSI datasets requires image registration and aims to combine multiple adjacent 2D MS images of consecutive cuts of a biological sample to a single 3D MS image volume. Besides the critical issue of serial tissue sectioning, preparation, and acquisition, the processing and handling of 3D MSI data has been regarded as a huge bottleneck so far [6].

Another big task requiring image registration is in multi-modal set-ups for the combined analysis of MSI and another imaging modality such as microscopy images of immunofluorescence or haematoxylin-eosin (H&E)-stained tissue sections. Challenges emerge from cases where adjacent tissue sections are used that may be distorted, and/or images containing no corresponding features due to the different imaging modalities.

Generally, the goal of image registration is to spatially align 2 adjacent images, commonly referred to as fixed image *I*^F^ and moving image *I*^M^. Registration aims to find the parameters of a transformation that maps *I*^M^ onto *I*^F^. Methods exist that are based on reference points highlighting landmarks in both the fixed and moving image, as well as image-based methods that try to minimize an image-based similarity metric by iterative optimization. A distinction is made between rigid and deformable registration approaches, with the task deciding which method is chosen or whether both are used in combination. M^2^aia integrates the elastix [24] toolkit for registration tasks.

#### 3D Image reconstruction

M^2^aia provides an interactive solution for performing 3D reconstruction. To create a 3D MSI volume, M^2^aia aligns consecutive slices to each other, applying subsequent rigid and deformable image-based registration steps in a fully automated way. This workflow has some technical challenges: (i) the provisioning of multiple MSI data at the same time, (ii) the choice of which image content is used for registration, and (iii) the parameterization of the rigid and deformable registration steps.

To address the first challenge, M^2^aia allows multiple MSI images to be loaded and makes those accessible for further processing steps. Here, the already described lazy loading mechanism shows its advantages. Regarding the second challenge, it is necessary to find feature-rich ion images (*m*/*z* ranges) that capture corresponding tissue structures in the moving and fixed image. If no such ion images are known beforehand, it is possible to search interactively for ion images. M^2^aia supports this by plotting overview spectra and the result of a peak-picking action. For the third challenge, M^2^aia offers an in-app editor to modify a template parameter file that is passed to elastix. In Fig. 4 the 3D reconstruction of the publicly accessible 3D dataset accompanying this article is exemplarily illustrated. In Use-case 2 we further elaborate on M^2^aia’s 3D reconstruction and multi-modal image registration capabilities.

#### Multi-modal image registration

Image-to-image registration of multiple image sources can help to elucidate the relationship of observations that can only be detected in different image domains. It is not guaranteed that there is a large correspondence of image features in different domains, especially between MSI and other, e.g., optical, imaging methods. Owing to this fact, similarity metrics based on mutual information are used for image comparison.

For the case of multi-modal MSI, it is necessary to choose appropriate image contrasts in the respective MSI domains of the moving and the fixed image. A major advantage of M^2^aia is that it allows the handling and visualization of individual mass axes of multiple MS images in a common coordinate system. This enables the user to select ion images with high mutual information in an interactive environment.

### Multi-modal 3D MSI dataset

To demonstrate the capabilities of M^2^aia we make a lipid 3D and a peptide 3D MSI dataset publicly available [36] together with this article. The dataset consists of 10 consecutive cuts of brain tissue taken from an APP NL-G-F mouse model [41]. Briefly, the sample was cut with a thickness of 10 $\mu {\rm m}$  and sections were placed on a single Bruker indium-tin oxide slide. Subsequently, lipid and peptide MALDI–time-of-flight (TOF) MSI acquisitions were made. In between the data acquisition for lipids and peptides, the matrix and most of the lipids remaining on the tissue sections were washed away before the peptide acquisition protocol was applied. The lipid and peptide datasets share a common lateral resolution of 20  $\mu {\rm m}$ and a spot size of 20 × 20 $\mu {\rm m}$. The lipid 3D and peptide 3D datasets are published here for the first time. All experiments were approved (No.142/2015) by the Ethics Committee on Animal Experimentation of the University of Leuven.

#### Lipid MSI

Measurement was done on a Rapiflex MALDI-TOF MS (Bruker Daltonics, Bremen, Germany) in reflector positive mode with *m*/*z* 600–1,800 using FlexImaging 5.0 software (Bruker Daltonics, Bremen, Germany). In brief, the acquisition method was calibrated using polyalanine calibration standard and quadratic calibration. Two hundred laser shots at 10 kHz repetition speed were accumulated for each raster spot.

#### Peptide MSI

Prior to peptide MSI, tissue sections were delipidated using the washing procedure by Yang and Caprioli [17]: 70% ethanol (30 s), 100% ethanol (30 s), Carnoy fluid (60/30/10 ethanol/chloroform/acetic acid v/v/v) (120 s), 100% ethanol (30 s), ddH2O (30 s), and 100% ethanol (30 s). Peptide MSI was performed on a Rapiflex MALDI-TOF MS (Bruker Daltonics, Bremen, Germany) in positive linear mode with *m*/*z* 2,000–10,000 using FlexImaging 5.0 software (Bruker Daltonics, Bremen, Germany) as described elsewhere [7].

MSI features describing the distribution of several occurrences of β-amyloids in a peptide dataset of the same specimen, which are related to the chosen APP NL-G-F mouse model and Alzheimer disease, are described in a separate publication by Enzlein et al. [7].

### Use-case 1: N-linked glycan *m/z* candidate detection

We reanalyzed a publicly available N-linked glycan MALDI-TOF dataset [34,42] to demonstrate the applicability of M^2^aia. A similar study was done by Föll et al. [29] using the application framework Galaxy, which is designed for server-side processing. The dataset is available in the PRIDE repository with accession code PXD009808. The data were published by Gustafsson et al. [34] and used to examine an automated sample preparation approach for MALDI-TOF/TOF imaging of N-linked glycans on formalin-fixed paraffin-embedded (FFPE) murine kidney tissue [34,42]. PNGase F was printed on 2 FFPE kidney sections to release N-linked glycans from proteins. A part of the third kidney was covered with N-glycan calibrants and another part with buffer to serve as a control. Imaging was performed with a spatial resolution of 100  $\mu {\rm m}$.

**Figure 6:**
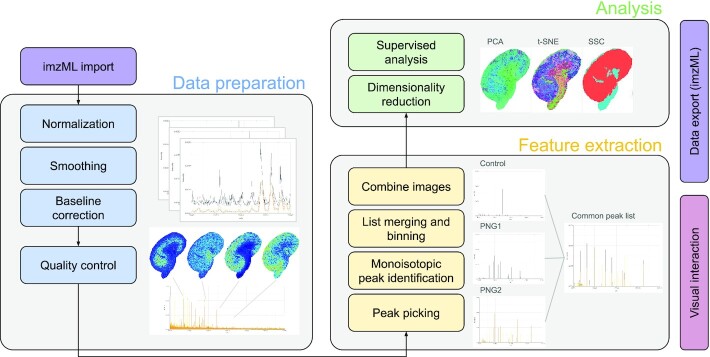
Typical steps to transform an MSI dataset into discriminating information in M^2^aia, exemplified by images from the reanalysis process of an N-glycan MALDI-TOF MSI dataset (Use-case 1).

Using M^2^aia, we loaded 3 datasets (PNG1, PNG2, and control; in total ∼6.4 GB; skipping the calibrant area) and applied TIC normalization, Savitzky-Golay smoothing, and Top-Hat baseline correction (Fig. 6, Data preparation). Peak picking with monoisotopic peak identification was applied to the mean spectrum of each image, respectively. The peak results of the datasets were combined into a common peak list. Peak binning was applied to remove duplicates, resulting in a list of 107 *m*/*z* (candidate) peaks (Fig. 6, Feature extraction).

To demonstrate how M^2^aia can be used in combination with other tools, we exported the processed data as a single imzML file to continue the processing with Cardinal (2.6.0) [43]. Providing the list of common peak features during the export process allows the data to be stored in continuous centroid format. Using Cardinal, we compare the 2 PNGase F–treated kidney tissue sections with the control tissue section for the identification of discriminant *m*/*z* candidates that are potentially related to N-linked glycans. N-linked glycan *m*/*z* candidates were selected by the supervised spatial shrunken centroids (SSC) algorithm [44,45] (Fig. 6, Analysis). Therefore, all pixels were separated into the classes “treated” (for PNG1 and PNG2) or “untreated” (for Control). By mapping the *treated* features selected by the SSC to the original publication of Gustafsson et al. [34], we could identify 16 N-linked glycan–related *m*/*z* candidates, as listed in Table 2. We calculated PCA images including the first 3 principal components and a t-SNE image (Fig. 5) based on the common peak list in M^2^aia.

**Table 2.** Potential *m*/*z*-candidates related to N-linked glycans

ID No.	*m*/*z*	t-Statistics	Error (ppm)		LC-MS/MS M+NA+	Composition
			This article	Föll et al. [29]		
1	1,257.4730	63.79	50	51	1,257.41	(Hex)_2_+(Man)_3_(GlcNAc)_2_
2	1,419.5177	60.62	33	59	1,419.47	(Hex)_3_+(Man)_3_(GlcNAc)_2_
3	1,743.6281	54.50	33	67	1,743.57	(Hex)_5_+(Man)_3_(GlcNAc)_2_
4	1,905.6748	50.93	23	30	1,905.63	(Hex)_6_+(Man)_3_(GlcNAc)_2_
5	1,581.5697	50.58	25	61	1,581.53	(Hex)_4_+(Man)_3_(GlcNAc)_2_
6	2,304.8962	47.12	28	36	2,304.83	(Hex)_2_(HexNAc)_3_(deoxyhexose)_3_+(Man)_3_(GlcNAc)_2_
7	1,850.7140	46.86	34	34	1,850.65	(Hex)_1_(HexNAc)_3_(deoxyhexose)_1_+(Man)_3_(GlcNAc)_2_
8	1,809.6975	45.44	37	52	1,809.63	(Hex)_2_(HexNAc)_2_(deoxyhexose)_1_+(Man)_3_(GlcNAc)_2_
9	2,158.8425	38.62	33	54	2,158.77	(Hex)_2_(HexNAc)_3_(deoxyhexose)_2_+(Man)_3_(GlcNAc)_2_
10	1,663.6324	35.92	37	58	1,663.57	(Hex)_2_(HexNAc)_2_+(Man)_3_(GlcNAc)_2_
11	1,485.5967	33.83	44	63	1,485.53	(HexNAc)_2_(deoxyhexose)_1_+(Man)_3_(GlcNAc)_2_
12	1,688.6586	31.59	28	62	1,688.61	(HexNAc)_3_(deoxyhexose)_1_+(Man)_3_(GlcNAc)_2_
13	2,012.7717	26.88	30	37	2,012.71	(Hex)_2_(HexNAc)_3_(deoxyhexose)_1_+(Man)_3_(GlcNAc)_2_
14	1,647.6444	26.83	45		1,647.57	(Hex)_1_(HexNAc)_2_(deoxyhexose)_1_+(Man)_3_(GlcNAc)_2_
15	2,816.1882	24.31	63	63	2,816.01	(Hex)_3_(HexNAc)_4_(deoxyhexose)_1_+(Man)_3_(GlcNAc)_2_
16	2,067.7292	19.00	28	43	2,067.67	(Hex)_7_+(Man)_3_(GlcNAc)_2_

By the reanalysis of the data published by Gustafsson et al. [34] in M^2^aia, we were able to identify a set of 16 discriminating *m*/*z* features (col. 2) obtained from the MSI data and mapped it to the LC-MS/MS experiment [34] (col. 6) for the treated kidney sections. Errors are listed for M^2^aia (col. 4) and Föll et al. [29] (col. 5) for comparison. Compositions [34] of the corresponding *m*/*z* features are reported in col. 7. t-Statistics of the supervised spatial shrunken centroids algorithm are reported in col. 3. Column 1 reports the identifier (ID) for sorted features by descending t-statistics. Hex: hexose; Man: mannose; GlcNAc: N-acetyl-D-glucosamine; HexNAc: N-acetyl-D-hexosamine.

For reproducibility purposes, protocols of the interactive steps from loading to exporting [46] and for dimensionality reduction [47] are available. The R-based processing of the intermediate results is available as a CodeOcean capsule [48, 49]. An additional CodeOcean capsule implements the described workflow as a command-line application [50,51], demonstrating the possibility of developing M^2^aia-based applications for batch-processing and porting them to a server infrastructure.

### Use-case 2: Multi-modal 3D image reconstruction

As mentioned before, the dataset published together with this article consists of 10 consecutive brain slices of an APP NL-G-F mouse model, imaging both lipid and peptide features (in total ∼80 GB in size). The objective of the use-case described in the following is to demonstrate the applicability of M^2^aia for mono- and multi-modal 3D image reconstructions by showing how to embed the peptide information into the lipid structural context in Three dimensions.

To demonstrate mono-modal 3D reconstruction, all 10 slices of the lipid and the peptide datasets were loaded into M^2^aia, respectively, and used for slicewise reconstruction of 3D image stacks. For multi-modal 3D-reconstruction, the peptide dataset was pairwise registered with the respective lipid slices of the previously reconstructed 3D lipid image stack.

Each of the 10 lipid imzML binary files is ∼4.9 GB on disk and each of the 10 peptide imzML binary files is ∼2.8 GB. Loading and initialization of a single lipid image into M^2^aia took a mean of 3.26 ± 0.67 seconds for the lipid data and 2.06 ± 0.45 seconds for the peptide data. During the initialization, maps of TIC normalization factors and the TIC-normalized overview spectra are created for each dataset.

Successful image-based registration requires images that are rich and similar in structural features. This can be done in M^2^aia by fast and interactive exploration of ion images. For the example data, structure-rich images in the lipid dataset were found at *m*/*z* 865 ± 0.65 Da and for the peptide dataset at *m*/*z* 2,250 ± 50 Da. For a rough initial alignment of considerably rotated tissue sections, we took advantage of M^2^aia’s capability to interactively rotate the slices by ±15° around the center. Additionally, the non-tissue areas were removed from the ion image generation process by segmentation of the respective areas, using the segmentation tools provided by M^2^aia.

**Figure 7:**
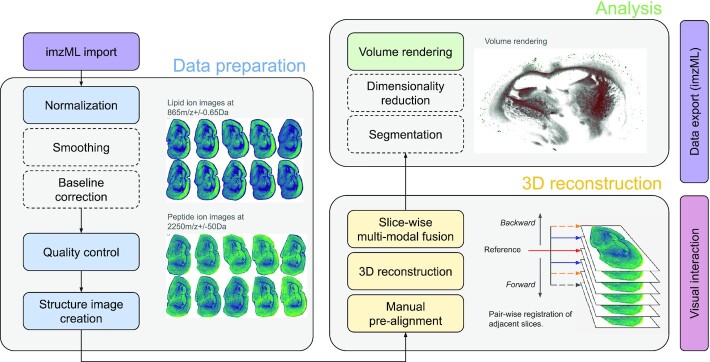
Steps for 3D reconstruction of consecutive MSI image slices in M^2^aia, exemplified by images from the 3D reconstruction and registration of the publicly available MALDI-TOF lipid and peptide dataset. Dashed boxes are possible additional processing steps that were not applied to the data shown.

For mono-modal 3D reconstructions, a reference slice was first selected in the corresponding M^2^aia plugin from the list of ordered slices. Starting from this reference slice, adjacent slices were automatically aligned to each other by rigid and deformable image-based registration. The process is applied to the image stack in both downward and upward directions (see 3D reconstruction section of Fig. 7).

Rigid registration is based on a multi-resolution registration strategy (Gaussian pyramid with 3 levels and downsampling factors of 4, 2, 1). Advanced Mattes mutual information [24] is used as metric for the optimization of a Euler transformation using linear interpolation and 250 iterations.

For the subsequent deformable registration steps, the same multi-resolution scheme and metric are applied. As deformable transformation, a recursive B-spline transformation is used with final grid spacing on the original resolution set to 0.8 mm, with scaling factors per pyramid level of 2, 1.5, and 1, respectively. Interpolation is performed by third-order B-splines. The optimization is run for 750 iterations. Figure 7 summarizes the workflow.

**Figure 8:**
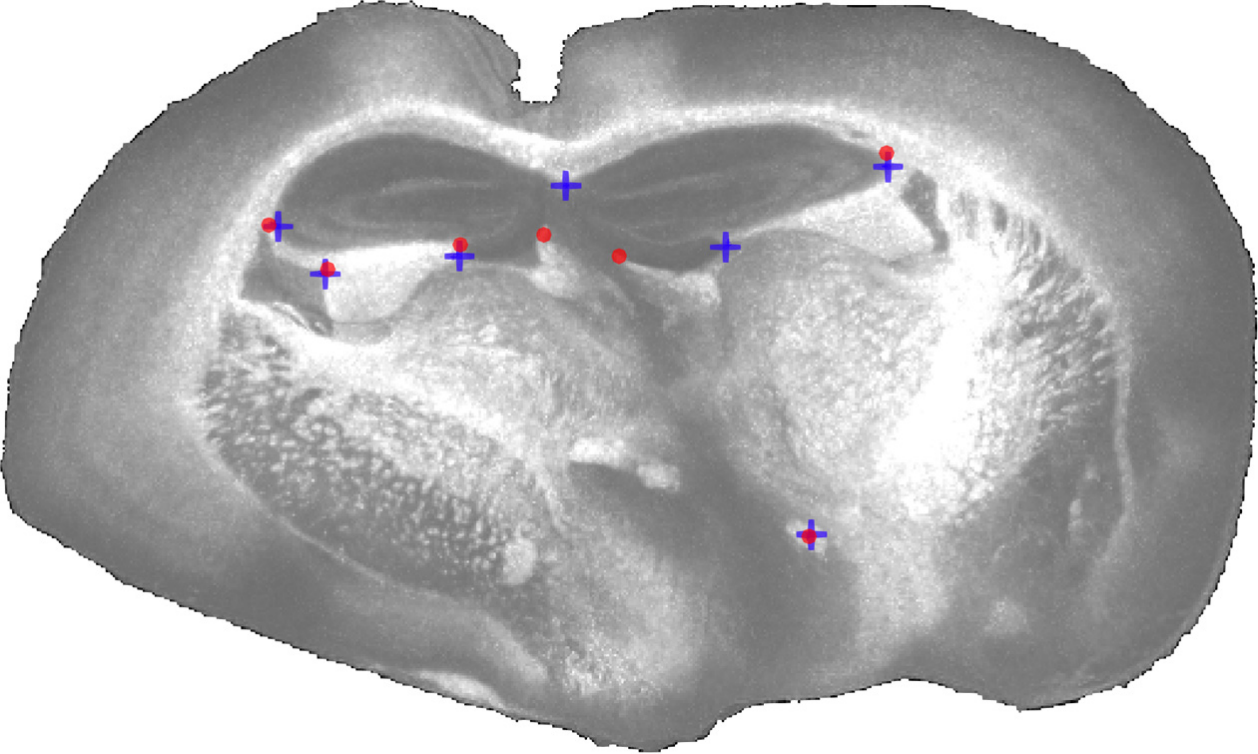
Maximum intensity projection of the multi-modal 3D reconstructed dataset at *m*/*z* 865 ± 0.65 Da. Peptide (blue crosses) and lipid (red circles) reference points are shown for the mid-slice of the stack.

To quantify the accuracy of the registration, we used M^2^aia to interactively select 7 reference points in each slice and in both modalities independently (a subset of points share a common anatomical location in both modalities), resulting in 70 reference points per set. In Fig. 8 the reference points of both modalities are shown in context of the reconstructed lipid dataset for the mid-slice of the stack.

For the mono-modal 3D reconstructions of the lipid (see Fig. 4) and peptide datasets, we obtained a mean registration error of 28 ± 8 and 35 ± 5 $\mu {\rm m}$, respectively, and for the multi-modal reconstruction a mean reconstruction error of 39 ± 4 $\mu {\rm m}$.

A protocol [52] showing how to perform the interactive steps in M^2^aia of the workflow as described above is available on protocols.io. Additional File 1 shows a rotating view of the volume visualization of the reconstructed data.

## Discussion

M^2^aia’s multi-threading and lazy loading concepts enable memory-efficient exploration of datasets that are far larger than the system’s actual working memory (Fig. 3). As shown in Table 1, loading a 44.2-GB dataset requires <500 MB of RAM. This allows even complex MSI analysis tasks to be performed on standard PCs. We demonstrate this for *m*/*z* candidate detection on an N-linked glycan MALDI-TOF dataset and 3D multi-modal registration of a lipid and peptide dataset. All steps of the use-cases were performed on a laptop computer with 16 GB of RAM.

M^2^aia's GUI is intended to remove existing barriers related to performing the steps of an MSI study. Additionally, all features of the M^2^aia programming API can also be used to create command-line applications. This is beneficial for batch processing of huge databases of MS images. In this case the interactive M^2^aia GUI can help us to understand the implications of each processing step during development and quality control.

In addition to interactive processing with M^2^aia, it is possible to create memory-efficient command-line applications that also benefit from the multi-threading and lazy loading approaches. By implementing command-line applications within an OS-level virtualization (e.g., Docker) a scalable, distributable, and reproducible server-side MSI processing environment can be created and integrated with established workflow tools such as Galaxy.

Image-to-image registration requires structure-rich images that include common characteristic features between tissue slices or, for the multi-modal case, between modalities. This may require a user-driven search for structure-rich images across the *m*/*z* dimension, which is facilitated by M^2^aia's fast and interactive ion image generation. Because different masses are intrinsically registered, it is irrelevant whether the structures visible in an image are meaningful entities or imaging/normalization artefacts. To demonstrate this, an unusually wide mass range of 50  Da at *m*/*z* 2,250 was chosen to generate a structure-rich image. This was successfully used in Use-case 2 for the 3D reconstruction. Disabling TIC normalization for the same *m*/*z*-range leads to a noisy image without structures, not usable for image-based registration—suggesting that the contrast is actually caused by a TIC normalization artefact.

Another challenge for purely automatic image registration approaches is significantly misaligned, especially heavily rotated, or, even worse, flipped images. The interactive environment of M^2^aia makes it possible to quickly obtain a rough pre-alignment of the images that is sufficient as initialization for subsequent automatic refinement. With the possibility to edit the elastix parameter file, M^2^aia offers unrestricted access to the full potential of the elastix toolkit to enable problem-specific customization of image registration.

Evaluation of registration results is yet another task that requires interaction. It is performed either qualitatively by visualization methods (like blending or checkerboard visualization) or quantitatively by comparing corresponding landmarks or, less accurately, segmentations. Both methods typically require interactive tools, e.g., to select the appropriate parameters for visualization, to define corresponding landmarks, or to perform (or at least verify) segmentations. MITK, the toolkit that M^2^aia is based on, offers such tools. Future releases of M^2^aia will make them more easily accessible for MSI data.

With rare exceptions, transforming an image to another coordinate system requires interpolation of image data. If interpolation is applied to spectral data, the interpolated spectra must be interpreted with caution. To avoid possible misinterpretation of interpolated spectra, M^2^aia currently calculates only interpolated ion images and allows the transformation parameters to be stored for use together with the unmodified MSI data. To avoid interpolation of spectra in a multi-modal registration task with MSI and non-MSI data, the MSI image domain should be used as the fixed image domain.

Multi-modal MS imaging refers to approaches with different MSI contrasts (like lipid and peptide MS imaging), as well as combined MSI and non-MS imaging methods, e.g., MSI combined with microscopy. M^2^aia’s capabilities for the former scenario were demonstrated in Use-case 2. Combining MSI and microscopy is a common multi-modal MSI experiment with its own challenges in interactive visualization. Owing to the high lateral resolution of microscopy images, memory-efficient handling of microscopy datasets requires pyramidal and tiled storage approaches. To enable this in M^2^aia we are currently developing an interface for reading whole-slide images by utilizing the OpenSlide library [53]. This will be part of an upcoming release of M^2^aia.

## Conclusion

To our knowledge, M^2^aia is the first open-source application that provides interactive, fast, and highly memory-efficient access to multiple 2D/3D MS images at the same time. It offers all steps of a typical MSI signal processing work flow, responsive visual interaction in 2 and 3 dimensions, as well as image processing functions such as segmentation, and features deformable 3D reconstruction and multi-modal registration. Another unique feature is that fused data with individual mass axes can be visualized in a shared coordinate system. Furthermore, M^2^aia is an extensible framework allowing the development of custom MSI analysis pipelines.

## Data Availability

- M^2^aia binaries for Windows and Linux [54].
- M^2^aia source code [55].
- The 3D reference datasets by Oetjen et al. [5] used to generate the results of the timing experiments are available on the MetaboLights repository with accession code MTBLS176.
- Supporting data of Use-case 1: N-linked glycan MALDI MSI datasets by Gustafsson et al. [42] are available in the PRIDE repository with accession code PXD009808.
- Supporting protocol of Use-case 1: interactive steps from loading to exporting [46].
- Supporting protocol of Use-case 1: dimensionality reduction [46].
- Supporting capsule of Use-case 1: command-line application based processing [50].
- Supporting capsule of Use-case 1: R-based processing [48].
- Supporting data of Use-case 2: multi-modal 3D lipid and peptide mouse brain MSI data data have been deposited to the GigaDB repository [36].
- Supporting protocol of Use-case 2: interactive steps of 3D image reconstruction [52].

## Availability of Supporting Source Code and Requirements

- Project name: M^2^aia—MSI applications for interactive analysis in MITK
- Project home page: https://www.github.com/jtfcordes/m2aia
- Operating systems: Windows and Unix
- Programming language: C++, CMake, and R
- Other requirements: The project is based on the MITK snapshot/2020-12-21 [56] (required for compiling) and elastix (RRID:SCR_009619) v5.0.0 binaries [57] (required for running)
- License: BSD
- RRID:SCR_019324
- biotools:m2aia

## Additional Files

Additional File 1: Volume visualization of the multi-modal 3D reconstruction. A rotating view was generated for the result of Use-case 2, showing a volume visualization of *m*/*z* 864 ± 0.65 Da in blue-violet and *m*/*z* 4070 ± 5 Da in green.

## Ethics, Consent, and Permissions

All experiments were approved (No.142/2015) by the Ethics Committee on Animal Experimentation of the University of Leuven.

## Abbreviations

API: Application Programming Interface; CPU: central processing unit; DR: dimensionality reduction; GUI: graphical user interface; H&E: hematoxylin-eosin; ibd: imaging binary data; LC-MS/MS: liquid chromatography tandem mass spectrometry; JIP: Joint Imaging Platform; M^2^aia: MSI applications for interactive analysis in MITK; MALDI: matrix-assisted laser desorption/ionization; MS: mass spectrometry; MSI: mass spectrometry imaging; mz or *m*/*z*: mass-to-charge ratio; OS: operating system; PCA: pricipal component analysis; ppm: parts per million; PRIDE: proteomics identifications; RAM: random access memory; SSC: spatial shrunken centroids; SSD: Solid-State-Drive; t-SNE: t-stochastic neighbourhood embedding; TIC: total ion count/current; TOF: time of flight.

## Competing Interests

The authors declare that they have no competing interests.

## Funding

This work was funded by the German Federal Ministry of Research (BMBF) as part of the Innovation Partnership M^2^Aind, project M^2^OGA (13FH8I02IA) within the framework FH-Impuls. The article processing charge was funded by the Baden-Württemberg Ministry of Science, Research and the Arts (MWK) in the funding programme Open Access Publishing.

## Authors’ Contributions

J.C. developed and tested M^2^aia and created the use-cases and protocols. T.E. and C.M. prepared and acquired the lipid and peptide 3D data that were made publicly available. T.E. and M.H. helped test the application. M.H. contributed to the development of the visualization strategies. I.W. and C.H. contributed to the conceptualization and funding acquisition. S.E. contributed to the conceptualization. J.C. and I.W. wrote the manuscript. All authors critically read and approved the manuscript’s contents.

## Supplementary Material

giab049_GIGA-D-21-00033_Original_Submission

giab049_GIGA-D-21-00033_Revision_1

giab049_GIGA-D-21-00033_Revision_2

giab049_Response_to_Reviewer_Comments_Original_Submission

giab049_Response_to_Reviewer_Comments_Revision_1

giab049_Reviewer_1_Report_Original_Submission Chris Armit -- 3/2/2021 Reviewed

giab049_Reviewer_2_Report_Original_Submission Liam McDonnell -- 3/9/2021 Reviewed

giab049_Reviewer_3_Report_Original_Submission Nathalie Agar -- 3/16/2021 Reviewed

giab049_Reviewer_4_Report_Original_Submission Nathan Heath Patterson -- 3/22/2021 Reviewed

giab049_Reviewer_4_Report_Revision_1 Nathan Heath Patterson -- 5/20/2021 Reviewed

giab049_Supplemental_Video
