# Supplementary material for: M2aia—Interactive, fast, and memory-efficient analysis of 2D and 3D multi-modal mass spectrometry imaging data
Source: Gigascience. 2021 Jul 20;10(7):giab049. doi: 10.1093/gigascience/giab049 (PMC8290197; doi:10.1093/gigascience/giab049)
Supplement: giab049_GIGA-D-21-00033_Revision_1 [file giab049_giga-d-21-00033_revision_1.pdf]

GigaScience

M²aia - Interactive, fast and memory efficient analysis of 2D and 3D multi-modal mass spectrometry imaging data

--Manuscript Draft--

**Manuscript Number:**	GIGA-D-21-00033R1
**Full Title:**	M²aia - Interactive, fast and memory efficient analysis of 2D and 3D multi-modal mass spectrometry imaging data
**Article Type:**	Technical Note
**Funding Information:**	Bundesministerium für Bildung und Forschung (13FH8I02IA) — Mr. Carsten Hopf
**Abstract:**	Background: Mass spectrometry imaging (MSI) is a label-free analysis method for resolving bio-molecules or pharmaceuticals in the spatial domain. It offers unique perspectives for the examination of entire organs or other tissue specimens. Due to increasing capabilities of modern MSI devices, the use of three-dimensional (3D) and multi-modal MSI becomes feasible in routine applications - resulting in hundreds of gigabytes of data. To fully leverage such MSI acquisitions, interactive tools for 3D image reconstruction, visualization and analysis are required, which preferably should be open-source to allow scientists to develop custom extensions. Findings: We introduce M2aia (MSI applications for interactive analysis in MITK), a software tool providing interactive and memory efficient data access and signal processing of multiple large MSI datasets stored in imzML format. M2aia extends the Medical Imaging and Interaction Toolkit (MITK), a popular open-source tool in medical image processing. Besides the steps of a typical signal processing workflow, M2aia offers fast visual interaction, image segmentation, deformable 3D image reconstruction and multi-modal registration. A unique feature is that fused data with individual mass axes can be visualized in a shared coordinate system. We demonstrate features of M2aia by re-analyzing an N-glycan mouse kidney dataset and 3D reconstruction and multi-modal image registration of a lipid and peptide dataset of a mouse brain, which we make publicly available. Conclusions: To our knowledge, M2aia is the first extensible open-source application that enables a fast, user-friendly and interactive exploration of large datasets. M2aia is applicable to a wide range of MSI analysis tasks.
**Corresponding Author:**	Jonas Cordes, M.Sc.<br>Mannheim University of Applied Sciences: Hochschule Mannheim<br>Mannheim, Baden-Wurttemberg GERMANY
**Corresponding Author Secondary Information:**	
**Corresponding Author's Institution:**	Mannheim University of Applied Sciences: Hochschule Mannheim
**Corresponding Author's Secondary Institution:**	
**First Author:**	Jonas Cordes, M.Sc.
**First Author Secondary Information:**	
**Order of Authors:**	Jonas Cordes, M.Sc.<br>Thomas Enzlein, M.Sc.<br>Christian Marsching, Dr.<br>Marven Hinze, B.Sc.<br>Sandy Engelhardt, Jun. Prof. Dr.<br>Carsten Hopf, Prof. Dr.<br>Ivo Wolf, Prof. Dr.
**Order of Authors Secondary Information:**	

Powered by Editorial Manager® and ProduXion Manager® from Aries Systems Corporation

**Response to Reviewers:**	We sincerely thank the reviewers for their thoughtful comments and suggestions. We have improved the manuscript by carefully addressing the issues raised and provide a point-by-point response below. In the manuscript, we have highlighted major changes in blue to allow an easier tracking of the changes.<br><br>Reviewer #1: This excellent Technical Note describes M2aia (MSI applications for interactive analysis in MITK), a software tool enabling interactive signal processing and visualisation of Mass spectrometry imaging (MSI) datasets. As the authors explain, M2aia extends the well used Medical Imaging and Interaction Toolkit (MITK) (Nolden et al., Journal of Computer Assisted Radiology and Surgery. 2013 Jul.). Key features of the M2aia toolkit include image segmentation, dimensionality reduction, and elastix-based image registration. Importantly, M2aia supports read/write access to the open standard imaging format mz Markup Language (imzML) that is used for MSI data.<br><br>The authors highlight the use of M2aia through two use cases. The first use case explored N-linked glycan m/z candidate detection in formalin-fixed paraffin-embedded (FFPE) murine kidney tissue. The latter 3D reference dataset has previously been published in GigaScience (Oetjen et al., GigaScience, Volume 4, Issue 1, December 2015, s13742-015-0059-4), and the authors used this reference dataset to showcase dimensionality reduction and R-based processing of MSI data. The second use case was a study of multi-modal 3D image reconstruction in 10 consecutive brain slices, and this and was used to determine the spatial distribution of brain lipids and brain peptides in an APP NL-G-F mouse model. Importantly, the supporting APP NL-G-F mouse brain lipid and protein MALDI MSI data have been submitted to the GigaScience DataBase (GigaDB), as have the intermediate results of the N-glycan MALDI mouse kidney use case study.<br><br>The M2aia software tool is made publicly available on GitHub (https://github.com/jtfcordes/m2aia) where it has been ascribed an OSI-approved BSD-3-Clause License.I recommend this manuscript for publication in GigaScience.<br><br>>> Authors:<br>We thank the reviewer for highlighting the technical aspects in his summary of our work and appreciate the review.<br><br>Reviewer #2: The M2aia manuscript describes a set of routines that enable fast and memory efficient analysis of 2D and 3D multimodal mass spectrometry imaging datasets. The routines consist of data input, routine mass spectral processing of [time-of-flight-based] MALDI mass spectrometry MSI data, coregistration with histology or between MSI datasets, generation and visualisation of 3D MSI datasets, and basic dimensionality reduction methods (PCA and tSNE). From the perspective of MSI itself few of the routines are innovative in their own right, for instance the data reduction methods described here parallel those this reviewer published previously [J Am Soc Mass Spectrom. 2010 21:1969-78] and which have been adopted and refined by other groups since. Similarly the registration methods are also similar to those reported previously and which are correctly cited in the current manuscript. However many of those routines have remained academic exercises that have, much to this reviewer's chagrin, not been adequately disseminated to the wider community. The principal advantage of the work is that it provides powerful open source methods that the MSI community can adopt, exploit and improve further. So while it may be lamented that the routines seem overly focused on time-of-flight based MALDI MSI this reviewer would expect them to work very well for FTICR and Orbitrap data; similarly it may be correct that the choices for mass spectral processing or data reduction are rather limited in scope, and do not represent all of the tools that have been previously used, however that would be unfair. The authors have made an open source set of routines that enable multimodal 2D and 3D MSI, and which can be supplemented with additional routines/tools. It is a commendable effort that this reviewer is more than willing to recommend is published in its current status.<br><br>>> Authors:<br>We thank the reviewer for his thoughtful feedback and conclusions.<br><br>Reviewer #3: The authors present an interesting software tool for interactive visualization and analysis of mass spectrometry imaging data and its integration with

Powered by Editorial Manager® and ProduXion Manager® from Aries Systems Corporation

other imaging modalities. The paper is well presented and the authors' generosity to make it publicly available will be highly appreciated by the community. I very much appreciate the development of this tool, and below are a few suggestions that would be great to address:

>> Reviewer #3 – comment 1:
1- The authors mentioned "memory efficient" analysis, but it was not clear how they implement the memory efficient algorithm. Figure 2 would need some clarification. The results show data from two case studies with relatively smaller file size (data size of one case was reported 4.9 and 2.8 GB but the file size of the other case was not reported, please add). While in Table 1, the authors referred to much larger size data, how did they analyze 44.2GB of 3D mouse kidney data with only 235.1MB RAM usage? How is peak picking efficiently performed uploading full data? Please clarify this interesting point, especially since you highlight this as a problem in the field with continuous increase in data size.

>> Authors:
We thank the reviewer for pointing out that Figure 2 did not sufficiently clarify the implementation of the memory-efficient algorithms. Therefore, we have completely reworked Figure 2 and added new elements that highlight the data flow in memory-efficient processing tasks by utilizing multi-threading and lazy-loading. We also updated the caption to clarify the newly added aspects.

We added file size information to use-case 1. We want to clarify at this point (and tried to further emphasize this in the text), that in use-case 2 each single lipid and each single peptide image has a file size of about 4.9 GB and about 2.8 GB, respectively. The whole multi-modal 3D reconstruction process needs to process all ten lipid and all ten peptide files to M^{2}aia, accumulating to an overall data processing volume of about 80 GB.

To clarify memory usage, we added equation (1) to the Data Handling section (on page 4) that describes memory utilization during processing of a single MS image representation in M^{2}aia. We renamed some symbols to more consistently match the text with the content of figure 2: Set of spectra S and images I is now \hat{S} and \hat{I}, respectively. We added pixel type information to overview spectra and images.

Regarding that according to Table 1 only 435.1MB RAM was necessary to analyze 44.2GB of 3D mouse kidney data: 435.1MB RAM was sufficient, because for processing it is not necessary to load all spectral information, but only \hat{S} and \hat{I}. In this case, elements of \hat{I} are 3D images without full spectrum data, but offering memory-side single-ion, normalization, mask, and spectrum-index image data.

To show how "memory efficient" processing performs on current desktop system configurations and the largest reference dataset, we have added new timing experiment results to Table 1.

Regarding peak picking and other pixel-wise processing: This can be performed in a largely file-size-independent and memory efficient manner - as shown in the reworked Figure 2 as Data Processing Task (5) - by means of the multi-threading and lazy loading approaches.

We thank the reviewer for pointing out these important-to-clarify aspects.

>> Reviewer #3 – comment 2:
2- In the discussion, it would be great to discuss how you would envision the future development of this tool in light of analyzing big MSI data? For instance, if I have an imzML file of 100 GB or more, can I analyze it using the current version of this tool (how much memory would be needed?) or are you going to further extend it up for more scalable level?

>> Authors:
Regarding future developments, we have added remarks referring to our current developments to the last paragraph of the Discussion section on page 10.

Regarding the processing of imzML files of 100 GB or more: As we try to clarify above in our response to "Reviewer #3 - comment 1", the size of the data processed for use-case 2 was approx. 80 GB and thus in the order of magnitude of a dataset of 100 GB. For the calculation of how much memory is needed, please see also the response to "Reviewer #2 - comment 1".

>> Reviewer #3 – comment 3:
3- Image registration: there have been great efforts already done to automate the deformable registration between mass spectrometry imaging and other modalities. The authors covered key papers in the bibliography, but it would be valuable to shed more light either in the introduction or discussion to highlight the challenges, how the published methods addressed the challenges and how your tool benefited from it (if it did), and what are the existing challenges that still need to be addressed in this active research area.

>> Authors:
We added a more detailed discussion of image registration to the Discussion section on page 10.

Reviewer #4: The authors describe their software for MSI visualization and interactive analysis that is built on an existing image visualization and analysis framework, integrating the strength and development of the medical imaging community with MSI data.

Overall, this appears to be an excellent work and responds to the need and desires of the MSI community. While there have been multiple publications on 3D MSI, an open-source tool with a GUI for human-in-the-loop preprocessing and setup has yet to emerge and the described software can meet that need and provide such capabilities to non-bioinformatics experts.

>> Reviewer #4 - comment 1:
One issue that could be addressed is the use of the word "Fusion." Does this simply mean image registration between two images or is there an applied analysis where one gains information from another modality through data analysis like Nat Methods. 2015 Apr; 12(4): 366-372.

>> Authors:
We thank the reviewer for pointing this out. We have replaced "Fusion" by (image) registration. We also have done work on image fusion, but this is not yet part of the current version of M²aia and therefore not described in the paper.

>> Reviewer #4 - comment 2:
Can the authors comment on the output imzML and mention interpolation after registration? It is only briefly mentioned, and their registration methods, whether linear or non-linear will result in interpolation. It is possible the resulting "pseudo-spectra" generated after registration are no longer valid, or should at least be considered carefully, especially with regards to non-linear deformations that could potentially create large subsets of new pixels from interpolation.

>> Authors:
A discussion of this issue has been added to the Discussion section on page 10 (second last paragraph):

"With rare exceptions, transforming an image to another coordinate system requires interpolation of image data. If interpolation is applied to spectral data, the interpolated spectra must be interpreted with caution. To avoid possible misinterpretation of interpolated spectra, M2aia currently calculates only interpolated ion images and allows to store the transformation parameters for use together with the unmodified MSI data. To avoid interpolation of spectra in a multi-modal registration task with MSI and non-MSI data, the MSI image domain should be used as the fixed image domain."

>> Reviewer #4 - comment 3:
There is an important missing discussion around multi-modal MSI and microscopy data in this manuscript that could make it more powerful. This is by far the most common

	multi-modal MSI experiment and interactive visualization is not straightforward compared to strictly MSI datasets or even MSI-MSI multimodal data as shown in the manuscript. The authors are advantaged in their choice of toolkit as MSI data, while containing many spectral bands and typically stored as spectra rather than images, are similar to the medical images (MRI/ CT, etc.) in the spatial domain in terms of size (i.e., a few hundreds or thousands of pixels). However, the natural corollary to MSI in optical space is the whole slide image, which presents challenges in visualization (i.e., pyramidal, tiled storage that allow memory-efficient reading and fast rendering) that the authors have not demonstrated in their manuscript. It seems to exist based on a look through the code-base on GitHub and could be worth highlighting here in supplemental.<br><br>>> Authors:<br>We thank the reviewer for mentioning this important point. Interactive visualization of MSI data registered to Non-MSI data was not part of the 2021.02 release of M²aia which is described in the manuscript. We highlight in the discussion that MSI-to-Non-MSI registration and interaction/visualization are part of the current developments of M²aia and will be available in a future release.<br><br>>> Reviewer #4 - comment 4:<br>As a suggestion to enable adoption of the software, the authors may want to create a simple website that describes their software and has direct links to their binaries. Many non-informatics researchers may reach the GitHub repo splash page and never know where to actually download the software as the GitHub interface may seem intimidating.<br><br>>>Authors:<br>Thank you for this recommendation. We are going to setup a github.io page.
Additional Information:	
**Question**	**Response**
Are you submitting this manuscript to a special series or article collection?	No
**Experimental design and statistics**<br><br>Full details of the experimental design and statistical methods used should be given in the Methods section, as detailed in our Minimum Standards Reporting Checklist. Information essential to interpreting the data presented should be made available in the figure legends.<br><br>Have you included all the information requested in your manuscript?	Yes
**Resources**<br><br>A description of all resources used, including antibodies, cell lines, animals and software tools, with enough information to allow them to be uniquely identified, should be included in the	Yes

Powered by Editorial Manager® and ProduXion Manager® from Aries Systems Corporation

Methods section. Authors are strongly encouraged to cite Research Resource Identifiers (RRIDs) for antibodies, model organisms and tools, where possible.<br><br>Have you included the information requested as detailed in our Minimum Standards Reporting Checklist?	
**Availability of data and materials**<br><br>All datasets and code on which the conclusions of the paper rely must be either included in your submission or deposited in publicly available repositories (where available and ethically appropriate), referencing such data using a unique identifier in the references and in the "Availability of Data and Materials" section of your manuscript.<br><br>Have you have met the above requirement as detailed in our Minimum Standards Reporting Checklist?	No
If not, please give reasons for any omissions below.<br><br>as follow-up to "**Availability of data and materials**<br><br>All datasets and code on which the conclusions of the paper rely must be either included in your submission or deposited in publicly available repositories (where available and ethically appropriate), referencing such data using a unique identifier in the references and in the "Availability of Data and Materials" section of your manuscript.<br><br>Have you have met the above requirement as detailed in our Minimum Standards Reporting Checklist?	All data and code will be available, but its availability depends on this submission itself according to the rules of the used repositories:<br>- https://codeocean.com: the uploaded CodeOcean Capsules will be ready for review as soon as this submission has been started and submitted by CodeOcean system to the GigaScience editorial board.<br>- https://www.protocols.io: The protocols of use-case 1 and use-case 2 will refer to the GigaDB ID of the data described in the manuscript and uploaded together with this submission. Reviewers can access the protocols via the following private share link:<br>https://www.protocols.io/private/7CF597BA637963848F0AC06943ACF472<br>https://www.protocols.io/private/CE9860DD4F9858B40CAAA94A7D5D2198

"	

Manuscript

Click here to access/download;Manuscript;Cordes et al Feb 2021.pdf

GigaScience, 2021, 1–13

doi: xx.xxxx/xxxx
Manuscript in Preparation
Technical Note

TECHNICAL NOTE

M²aia – Interactive, fast and memory efficient analysis of 2D and 3D multi-modal mass spectrometry imaging data

Jonas Cordes[1,2], Thomas Enzlein[3], Christian Marsching[3], Marven Hinze[1], Sandy Engelhardt[4], Carsten Hopf[3] and Ivo Wolf[1]

[1]Faculty of Computer Science, Mannheim University of Applied Sciences, Paul-Wittsack-Straße 10, 68163 Mannheim, Germany and [2]Medical Faculty Mannheim, University Heidelberg, Theodor Kutzer-Ufer 1-3, 68167 Mannheim, Germany and [3]Center for Mass Spectrometry and Optical Spectroscopy (CeMOS), Mannheim University of Applied Sciences, Paul-Wittsack-Straße 10, 68163 Mannheim, Germany and [4]Working Group 'Artificial Intelligence in Cardiovascular Medicine' (AICM), University Hospital Heidelberg, Im Neuenheimer Feld 410, 69120 Heidelberg, Germany

Correspondence address. Ivo Wolf, Faculty of Computer Science, Mannheim University of Applied Sciences, Paul-Wittsack-Straße 10, 68163 Mannheim, Germany, E-mail: i.wolf@hs-mannheim.de

Abstract

Background: Mass spectrometry imaging (MSI) is a label-free analysis method for resolving bio-molecules or pharmaceuticals in the spatial domain. It offers unique perspectives for the examination of entire organs or other tissue specimens. Due to increasing capabilities of modern MSI devices, the use of three-dimensional (3D) and multi-modal MSI becomes feasible in routine applications – resulting in hundreds of gigabytes of data. To fully leverage such MSI acquisitions, interactive tools for 3D image reconstruction, visualization and analysis are required, which preferably should be open-source to allow scientists to develop custom extensions. **Findings**: We introduce *M²aia* (*MSI applications for interactive analysis in MITK*), a software tool providing interactive and memory efficient data access and signal processing of multiple large MSI datasets stored in imzML format. *M²aia* extends the *Medical Imaging and Interaction Toolkit* (MITK), a popular open-source tool in medical image processing. Besides the steps of a typical signal processing workflow, *M²aia* offers fast visual interaction, image segmentation, deformable 3D image reconstruction and multi-modal *registration*. A unique feature is that fused data with individual mass axes can be visualized in a shared coordinate system. We demonstrate features of *M²aia* by re-analyzing an N-glycan mouse kidney dataset and 3D reconstruction and multi-modal image *registration* of a lipid and peptide dataset of a mouse brain, which we make publicly available. **Conclusions:** To our knowledge, *M²aia* is the first extensible open-source application that enables a fast, user-friendly and interactive exploration of large datasets. *M²aia* is applicable to a wide range of MSI analysis tasks.
Key words: mass spectrometry imaging; *multi-modal; image registration;* image reconstruction; three-dimensional; interactive visualization

Introduction

Imaging of molecular information in the spatial domain enables insights into otherwise hidden conditions and metabolic

Compiled on: May 19, 2021.
Draft manuscript prepared by the author.

processes. Mass spectrometry imaging (MSI) represents a class of label-free and spot-wise spectrometry acquisition techniques [1] and plays already an important role in a wide range of bio-medical and industrial applications. It is a proven technique used for development of pharmacological agents [2] or for phenotyping of pathological tissue samples [3]. The spot-wise imaging process results in two-dimensional (2D) mass spectrometry (MS) imaging data, where in each pixel a spectrum is acquired representing the relative intensities of ionizable molecular compounds covering a wide range of mass to charge (m/z) ratios.

It is expected that the use of MSI techniques will increase dramatically in the future due to the development of faster acquisition techniques and improved accuracy of MSI devices [4]. The constantly growing file sizes of up to hundreds of gigabytes are a challenge for data processing, especially for interactive tasks like visualization and exploratory data analysis [5].

The research community can choose from a variety of software tools for MSI data [6]. Interactive exploration, analysis and processing of MSI data is not possible by the currently available open-source software solutions, as they are not designed for an interactive scenario where low latencies and an user-friendly graphical interface are desirable. On the other hand, interactive and fast feedback is important for successful experiments, allowing early intervention by inclusion of interesting or exclusion of invalid image regions into further analysis. If blackbox-like scripts are just applied, the traceability and quality of MSI data processing steps can suffer. Apart from that, exporting data of intermediate steps for visualization in external tools is time consuming error prone and difficult to handle, especially if running on a server infrastructure.

The emerging field of three-dimensional (3D) MSI [7, 8, 9, 10] offers new perspectives into the molecular structure of biological samples. 3D MS images can be generated by combining multiple adjacent 2D MS images of consecutive cuts of a biological sample to an MS image volume by co-registration (3D image reconstruction) [5, 11, 12].

Multi-modal MSI can refer to (i) imaging of lipids, peptides, and proteins on adjacent tissue sections or the same tissue section [13, 14, 15, 16] with intermediate removal of the matrix [17] (to apply different matrix preparation approaches) or (ii) the *registration* of MSI and another imaging modality like optical imaging [18, 19, 20, 21]. *Registration* of multi-modal data is challenging because of modality-dependent image contrasts. Especially difficult is to eliminate distortions caused by the preparation process, partial destruction in the original tissue morphology and spatially misplaced tissue sections (rotated or placed up side down). Since it is an emerging field of research, interactive environments for (semi-) automatic 3D reconstruction or multi-modal MSI experiments do not exist yet in an openly accessible and mature form. The currently most advanced commercial solution is SCiLS Lab (Bruker Daltonik GmbH, Bremen, Germany).

To recover the correct spatial relationship between corresponding images, registration methods need to be applied to perform mirroring, rigid or deformable image transformations. Open-source toolkits and command-line applications targeting intensity-based image registration are available, e.g. the Insight Toolkit (ITK) [22, 23] and elastix [24], and have been shown to be applicable to multi-modal image *registration* [19] and 3D MSI image reconstruction [12, 25].

2D/3D MSI results into large hyper-dimensional datasets that pose computational challenges to interactive visualization, exploration and analysis on commonly available software and hardware configurations. Software solutions for MSI are often limited by available RAM and time-consuming initialization procedures, which is especially true in the case of 3D image reconstruction where preferably all required 2D MSI datasets are accessible at the same time.

Aims

We created M^2aia (*MSI application for interactive analysis in MITK*), by integrating MSI support into the platform independent and open-source Medical Imaging Interaction Toolkit (MITK) [26]. M^2aia supports the major MS image-related processing tasks required in an MSI study within a single framework. These are illustrated in figure 1. M^2aia supports read/write access to MSI data in the open standard format *imaging mz Markup Language* (imzML) [27] supporting 2D/3D continuous and processed datasets, in addition to the (medical) image and image-related file formats supported by MITK. We developed M^2aia with four main goals in mind: (i) providing a complete set of memory-efficient and fast MSI utilities for interactive data visualization, signal processing and analysis, optionally usable for batch-processing; (ii) simultaneous handling of multiple, potentially multi-modal images with minimal memory overhead; (iii) support for user driven 3D MS image reconstruction and multi-modal image *registration*; (iv) the distribution of a community extendable and open-source code base.

In contrast to applications like Galaxy [28] that provide a server-side solution for non-interactive standardized processing of large MSI studies [29], M^2aia is designed to give a fast,

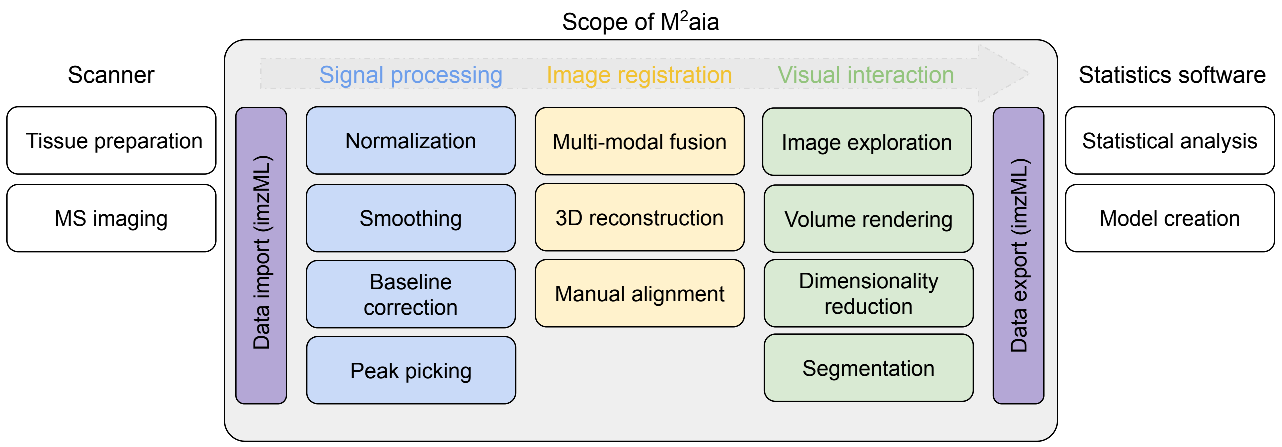

Figure 1. Commonly used MSI pipeline (left to right). The aim of M^2aia is to provide an interactive environment with support for all image-related tasks from data import to co-registration, exploration, segmentation and data export.

visual and interactive user response on commonly available hardware offering the above-mentioned features. All main features can be accessed by a user-friendly graphical interface and, therefore, no programming skills are required. Thus, the MSI extension of Galaxy [29] and M^2*aia* complement each other, offering different tools for a full stack open-source MSI working environment.

The Medical Imaging Interaction Toolkit (MITK) as an application and development backbone

MITK [26] is a popular open-source toolkit for development of interactive medical image processing applications. In essence, the MITK framework is characterized by (i) a flexible plugin-based user-interface and modular system environment; (ii) the capability of handling a wide range of 2D, 3D and 3D+t (medical) images and image-related data like surfaces, point sets and image segmentations; (iii) consistent interactive visualization of multi-planar reconstructions and 3D visualizations and (iv) a completely open-source code base built on top of ITK [22, 23], VTK [30, 31] and Qt [32]. MITK is a cross-platform C++ toolkit and officially supports Windows, Linux and macOS. A modular software structure allows the development of new applications decoupled from the main source tree of MITK. The Joint Imaging Platform (JIP) [33] provides methods to containerize MITK, allowing it to be hosted on a scalable server infrastructure, and offers Virtual Network Computing as an interface able to stream MITK to a Web browser.

Findings

M^2*aia* can be considered as an interactive open-source exploration and analysis application for MSI data with the capability of 3D image reconstruction and multi-modal image *registration* that is extensible by the community. Figure 3 gives an impression of the graphical user interface. In the following sections we introduce the main concepts and newly implemented features that are introduced by M^2*aia*. These features were compiled into several plugins targeting different aspects of MSI processing, including plugins for MSI import, export, signal processing, peak picking, multi-modal *registration* and 3D reconstruction. All MSI related plugins where build from scratch (except parts that incorporate third party technologies) and highly optimized for fast reaction times and low memory overhead. Thanks to the multi-platform paradigm of MITK, binaries for multiple operating systems, including Windows- and Unix-based systems, are available. In addition, the modular system of M^2*aia* allows the community a highly flexible development of plugins to add new functionalities and to develop command-line based applications for batch processing.

Finally, we provide two state-of-the-art use-cases. The first use-case demonstrates an m/z candidate detection task on publicly available N-linked glycan data [34] (available in the PRIDE repository [35] with accession code PXD009808). In the second use-case we elaborate on the 3D reconstruction capabilities of M^2*aia* using a multi-modal 3D MSI dataset of lipid and peptide acquisitions, which we make publicly available in the GigaScience respository, GigaDB [36]. Especially, the interactive way of visualizing 2D/3D multi-modal image *registration results* with individual mass axes in a shared coordinate system is a unique feature and, to our knowledge, M^2*aia* is the first open-source application that offers this kind of visual interaction.

Data handling

We implement support for the imzML file format (continuous-profile, continuous-centroid and processed-centroid with external data storage in an imaging binary data (*.ibd) file). Provided that MSI data can be converted into the imzML format, M^2*aia* is capable of processing data of various MSI devices, e.g. MALDI, DESI or SIMS.

One challenge in MSI is the handling of computing resources. Loading tens of gigabytes of data into the computer's memory is almost impossible without server-side processing capabilities. To face this challenge, we follow the strategy of *lazy loading*, resulting in minimal memory overhead and reaction times.

Internally in M^2*aia* the access to the imzML data is split into two steps. First, all necessary metadata M are read from the XML file (*.imzML), which contains both the image geometry information and all access information of the spectra (figure 2, *data processing task (1))*. Since the imzML files can become big XML structures (specially in 3D MSI several gigabytes), processing these files is very slow with conventional XML parsers. To overcome this problem we implemented an approach based on line-wise parsing reducing the consumed time to a minimum. *Second, an equal number of spectra is assigned to multiple threads, where each can read spectral data on demand (lazy loading) from the imaging binary data (*.ibd) container, using the meta data M from the first step (figure* 2*, data processing task marked with an asterisk). From this point on, the processing time depends on the user-defined signal processing and data processing task related steps*. Loaded binary data blocks (e.g., partial spectrum data) are discarded after processing. Only the resulting numbers, e.g. the values of scaling factors, overview spectra or pixels, are retained.

Additional to the above mentioned meta-data content M, a set of gray-scale image artifacts $\hat{I}$ and a set of overview spectrum artifacts $\hat{S}$ are kept in memory.

The set of image artifacts $\hat{I} = \{I^{ion}, I^{norm}, I^{index}, I^{mask}\}$ in-

Table 1. *Timing experiments on mobile and desktop systems using 3D reference data*

File Name	System	Size	Spectra/Depth	Parse meta-data (1)	Initialization (2)	Create ion image (3)	RAM usage
3D Mouse Kidney	A	44.2GB	1362830/7671	24.9s	26.27s	10.9s	435.1MB
3D Mouse Kidney	*B*	*44.2GB*	*1362830/7671*	*5.7s*	*2.1s*	*0.6s*	*435.1MB*
3D Mouse Pancreas	A	27.3GB	497225/13297	9.4s	13.14s	2.8s	292.4MB
3D OSCC	A	26.8GB	828558/7665	14.9s	13.4s	4.04s	323.5MB
Microbe Interaction 3D Timecourse	A	2.9GB	17672/40299	0.4s	0.9s	0.08s	231.8MB

Processing of 3D reference datasets published by Oetjen et al. [5] and available in the MetaboLights repository [MTBLS176]. The table lists the file names, number of spectra, depth of a spectrum and the average time in seconds and memory usage *including memory used by the graphical user interface* values of three manually repeated runs. Processes according to the procedures shown in figure 2. Applied signal processing includes total-ion-count (TIC) normalization. *System configuration A: mobile PC, Intel® Core™ i7-8750H CPU @ 2.20GHz 6-core processor, 16 GB physical memory and SSD. System configuration B: desktop PC, AMD® Ryzen 9 5900x CPU @ @ 3.7GHz 12-core processor, 32 GB physical memory and M.2 SSD.*

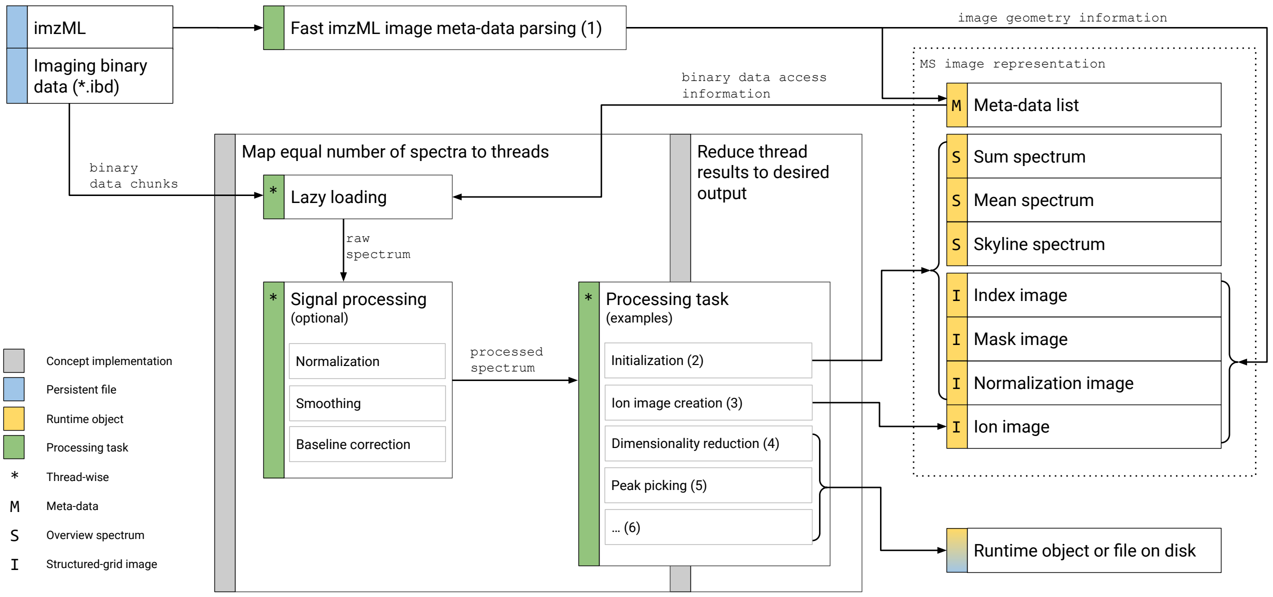

Figure 2. *Data flow for processing imzML files in M^2aia. Fast imzML image meta-data parsing (1) provides information about spectra and the image geometry. Spectra processing tasks (2)-(6) take advantage of the concept to assign equal parts of spectra to different threads. Each thread can trigger loading of spectra from disk (lazy loading). Signal processing can optionally be invoked. Processing task specific reduction of thread results produces the desired output. Spectrum data is discarded after processing and only the processing results are stored to keep memory consumption low.*

cludes I^{ion} as placeholder image for representation of ion images *(data type: double)*, I^{norm} as placeholder image of spectra related normalization factors *(data type: double)*, I^{index} an image with pixel positions according to the spectrum id in the imzML XML structure *(data type: int)* and I^{mask} a binary mask indicating where valid spectrum information is accessible *(data type: short)*. *All images have the same number of pixels* $|I|$.

The set of overview spectra $\hat{S} = \{S^{skyline}, S^{sum}, S^{mean}\}$ *includes* $S^{skyline}$ *as placeholder for the representation of the maximum spectrum,* S^{sum} *for the sum spectrum and* S^{mean} *for the mean spectrum over all spectra in the image. All overview spectra have the same spectral depth* $|S|$ *and data type double.*

$\hat{I}$ *and* $\hat{S}$ are generated once during the initialization of an MSI image in M^2aia and are held in memory as long as the MSI image is loaded. These artifacts require only a fraction of memory compared to loading the whole MSI spectra data. Data items are illustrated in figure 2.

The total memory E required for imaging and spectral data can be approximated with respect to the number of pixels of a placeholder image $|I|$, *spectrum depth* $|S|$ *and the sizes* δ^{type} *of the numeric types of the system in bytes. Additionally, during the multi-threaded processing, T threads are initialized, each of which at least allocates a single spectrum of size* $|S|$:

$$E = |I| * (2\delta^{double} + \delta^{int} + \delta^{short}) + (3 * |S| + T * |S|) * \delta^{double}. \quad (1)$$

Consequently, the memory required by M^2aia *is* $O(|I| + |S|)$ *and thus much lower than the total size of binary data which is* $O(|I| * |S|)$.

To test *the data handling of* M^2aia, we conducted performance measurements for the processing steps 1-3 (see figure 2) using the publicly available 3D reference datasets described in [5]. The list of the used reference datasets and the results are shown in table 1. The experiments were repeated three times each to obtain average processing times memory usage.

Signal processing methods

Signal processing is a fundamental and important step in MSI, since spectral analysis may be influenced by a wide range of factors including sample preparation, acquisition methods, chemical noise, analyte displacement and inconsistent intensities due to matrix or surface inhomogeneity, electronic fluctuations, or ionization effects [2]. Signal processing aims to reduce these influences. Therefore, M^2aia offers all steps of a typical signal processing workflow including (a) normalization, (b) noise reduction, (c) baseline correction and (d) peak picking methods.

a) Normalization/Calibration. To compensate pixel-to-pixel intensity variations, a spectrum S is normalized by

$$S(j) = S^{old}(j) * f_S^{-1}, \quad (2)$$

where $S^{old}(j)$ and $S(j)$ are the original and scaled intensities, respectively, f is the normalization value and j is the index related to the m/z position in a spectrum. If no normalization is applied, f is set to 1. Generated normalization maps are accessible for each image. As methods for determining f_S, M^2aia offers calculating total-ion-count (TIC), the median or the use of in-file normalization values (defined spectrum-wise in imzML, if available).

b) Baseline Correction. Most signal processing stages take advantage of baseline-corrected spectra. In MS, the baseline is the smooth curve offsetting the actual intensities. Baseline correction is generally performed by subtracting the estimated baseline from the intensity spectrum. Implemented is baseline estimation using the top-hat [37] or running median method.

c) Noise reduction. Spectrum-wise noise reduction is realized via the Savitzky & Golay filter [38] method.

d) Peak Picking. Peak picking refers to the detection of peaks in a spectrum and provides information about the m/z-values and corresponding intensities of the peaks. In M^2aia, peaks within

a spectrum are detected by finding local maxima above a certain noise level using a sliding window approach. The noise level is estimated by the median absolute deviation (MAD). Monoisotopic peaks can be identified by automatic Poisson peak harvesting [39].

Image interaction and processing

Key advantages of M^2aia are its interactive visualization and image-based processing methods. Visual-interaction with 2D and 3D data allows the exploration and perception of whole datasets. Dimensionality reduction methods can be applied to generate views that help to exploit the full potential of MSI. Methods for image segmentation can be used to restrict the analysis to relevant regions of interest. Image- and/or point-based registration methods enable performing 3D reconstructions of stacks of MSI images or image *registration* in multi-modal imaging setups. These concepts are described in more details below.

Data visualisation and interaction concepts. All images loaded to M^2aia are represented in a common virtual world coordinate system. This world space can be observed through multiple render windows that are showing different sliced views of the world space (multi-planar reconstructions, e.g. top-views and side-views of 3D stacks, tilted views are also possible). Complementary to the planar views, a 3D view is provided facilitating the perception of the distribution of structures in 3D space. Additional to the visualisation of pixel image data, M^2aia also support the incorporation of surfaces (e.g. created by segmentation of structures or loaded from files after external processing) and offers volume rendering for 3D image data (figure 4). Several color-maps are available and can be applied to images individually.

Image Segmentation. Image segmentation can be used for selecting regions or structures within an image. M^2aia offers semi-automatic segmentation tools for 2D/3D image data. Segmented areas can be statistically analyzed within M^2aia or exported to obtain insights into the local ion intensity distributions.

Dimensionality Reduction (DR). Various DR methods are available in M^2aia for the investigation of the high-dimensional MSI data. The most basic DR method is the extraction of ion images by an intensity transformation within an interactively selected window of m/z values. The intensity values within the window are transformed into a single representative value. Currently, M^2aia allows the calculation of mean, maximum, sum or median for this purpose.

More complex DR methods enable capturing features of the dataset distributed over many ion images at once. Principal component analysis (PCA) as a linear and t-stochastic neighborhood embedding (t-SNE) [40] as a non-linear DR method are available in M^2aia. For initialisation of the PCA or t-SNE, it is required to provide a finite set of ion images. Figure 6 illustrates the application of PCA and t-SNE to a centroid dataset.

Image Registration. Reconstruction of 3D MSI datasets requires image registration and aims to combine multiple adjacent 2D MS images of consecutive cuts of a biological sample to a single 3D MS image volume. Beside the critical issue of serial tissue sectioning, preparation and acquisition, the processing and handling of 3D MSI data has been regarded as a huge bottleneck so far [6].

Another big task requiring image registration is *in multi-modal setups*, for the combined analysis of MSI and another imaging modality like microscopy images of immunofluorescence or haematoxylin and eosin (H&E) stained tissue sections. Challenges emerge from cases, where adjacent tissue sections are used that may be distorted, and/or images containing no corresponding features due to the different imaging modalities.

Generally, the goal of image registration is to spatially align two adjacent images, commonly referred to as fixed image I^F and moving image I^M. Registration aims to find the parameters of a transformation that maps I^M onto I^F. Methods exist that are based on reference points highlighting landmarks in both the fixed and moving image, as well as image-based methods that try to minimize an image-based similarity metric by iterative optimization. A distinction is made between rigid and deformable registration approaches, with the task deciding which method is chosen or whether both are used in combination. M^2aia integrates the elastix [24] toolkit for registration tasks.

3D IMAGE RECONSTRUCTION. M^2aia provides an interactive solution for performing 3D reconstruction. To create a 3D MSI volume, M^2aia aligns consecutive slices to each other, applying subsequent rigid and deformable image-based registration steps in a fully automated way. This workflow has some technical challenges: (a) the provisioning of multiple MSI data at the same time, (b) the choice of which image content is used for registration and (c) the parametrisation of the rigid and deformable registration steps.

To address the challenge (a), M^2aia allows to load multiple MSI images and makes those accessible for further processing steps. Here, the already described "lazy loading" mechanism shows its advantages. Regarding (b), it is necessary to find feature-rich ion images (m/z ranges) that capture corresponding tissue structures in the moving and fixed image. If no such ion images are known beforehand, it is possible to search interactively for ion images. M^2aia supports this by plotting overview spectra and the result of a peak picking action. For (c), M^2aia offers an in-app editor to modify a template parameter file that is passed to elastix. In figure 4 the 3D reconstruction of the publicly accessible 3D dataset accompanying this paper is exemplarily illustrated. In use-case 2 we further elaborate on M^2aia's 3D reconstruction and multi-modal image *registration* capabilities.

MULTI-MODAL IMAGE *REGISTRATION*. Image-to-image registration of multiple image sources can help to understand the relationship of observations that can only be detected in different image domains. It is not guaranteed that there is a large correspondence of image features in different domains, especially between MSI and other, e.g. optical, imaging methods. Due to this fact, similarity metrics based on mutual information are used for image comparison.

For the case of multi-modal MSI, it is necessary to choose appropriate image contrasts in the respective MSI domains of the moving and the fixed image. A major advantage of M^2aia is that it allows the handling and visualization of individual mass axes of multiple MS images in a common coordinate system. This enables the user to select ion images with high mutual information in an interactive environment.

Multi-modal 3D MSI dataset

To demonstrate the capabilities of M^2aia we make a lipid 3D and a peptide 3D MSI dataset publicly available [36] together with this paper. The dataset consists of 10 consecutive cuts of brain tissue taken from a APP NL-G-F mouse model [41]. Briefly, the sample was cut with a thickness of 10 µm and sec-

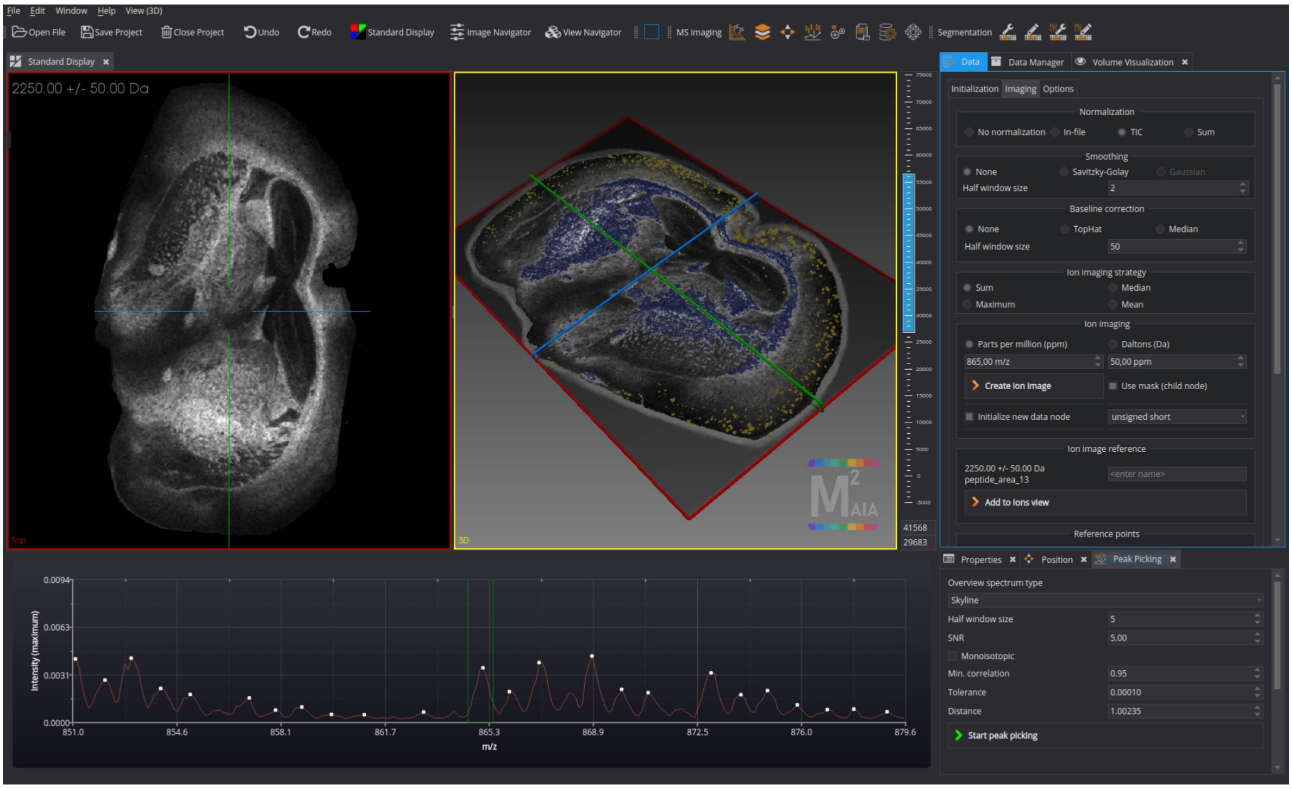

Figure 3. Graphical user interface. M^2aia introduces utilities for common MSI processing tasks and new workflows for 3D reconstruction and multi-modal image registration, extending the standard features of MITK [16] like semi-automated segmentation and 2D/3D visualization. Rendered images show a multi-modal 3D reconstructed volume of APP NL-G-F mice brain lipid and peptide MALDI-TOF datasets introduced in this paper. Volume rendering was applied to visualize high intensities of m/z 865±0.4 Da (blue) of the 3D reconstructed lipid dataset and m/z 4070±5 Da (yellow) of the 3D reconstructed peptide dataset. The plugin for controlling of the import and processing of MSI data is shown on the upper right side, the peak picking plugin on the lower right corner and the interactive spectrum view plugin on the lower left (here displaying the skyline spectrum of a lipid MS image).

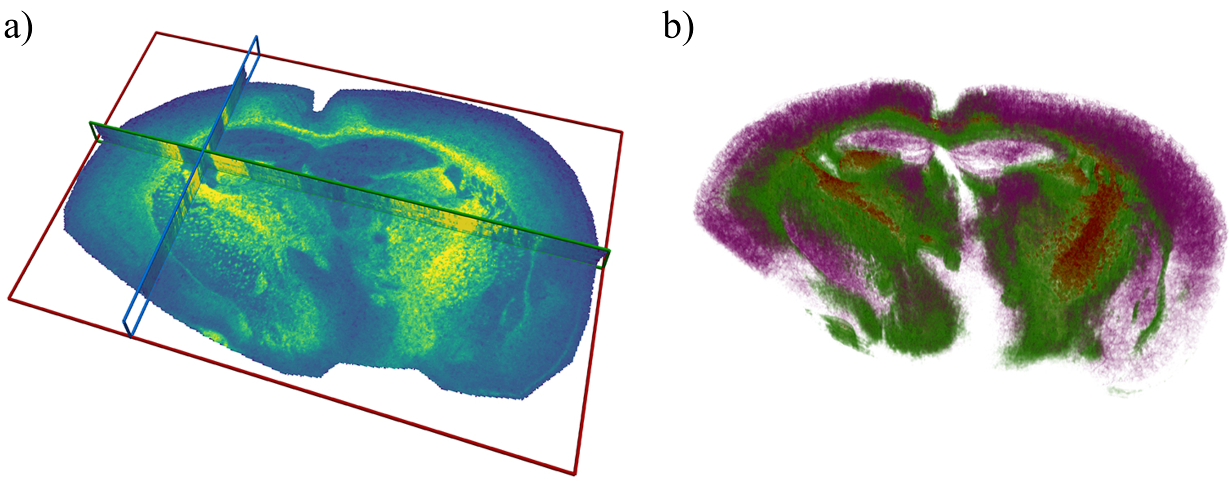

Figure 4. 3D reconstruction of lipid MALDI-MS TOF images of ten consecutive APP NL-G-F mouse brain tissue sections. a) Multi planar reconstruction of the 3D MS image showing ion images at m/z 865.05±0.1 Da. b) Volume visualization of different mass features. High intensities (green) and low intensities (red) in ion image at m/z 865.05±0.1 Da are visualized. Additionally, the cortex is highlighted by high intensities at m/z 868.76±0.1 Da (purple). Processing and visualization have been performed completely within M^2aia.

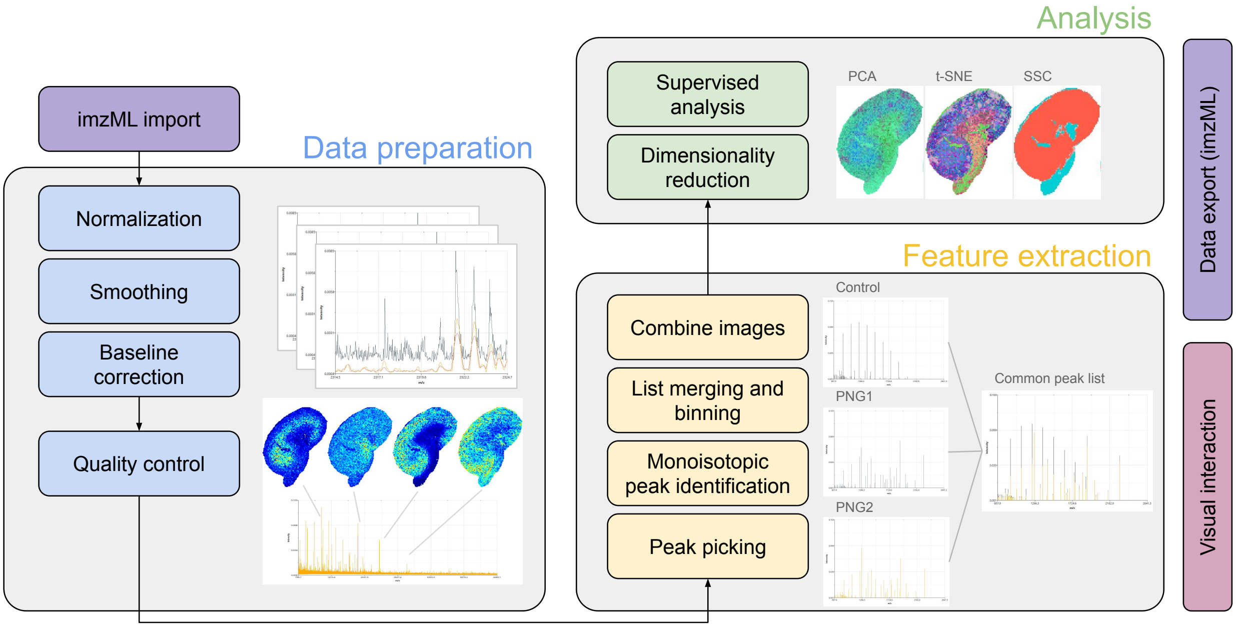

Figure 5. Typical steps to transform an MSI dataset into discriminating information in M^2aia, exemplified by images from the re-analysis process of a N-glycan MALDI-TOF MSI dataset (use-case 1).

tions were placed on a single Bruker indium-tin oxide (ITO) slide. Subsequently, lipid and peptide MALDI-TOF MSI acquisitions were made. In between the data acquisition for lipids and peptides, the matrix and most of the lipids remaining on the tissue sections were washed away before the peptide acquisition protocol was applied. The lipid and peptide datasets share a common lateral resolution of 20 µm and a spot size of 20 µm x 20 µm. The lipid 3D and peptide 3D datasets are published here for the first time. All experiments were approved (No.142/2015) by the Ethics Committee on Animal Experimentation of the University of Leuven.

Lipid MSI. Measurement was done on a Rapiflex MALDI-TOF MS (Bruker Daltonics) in reflector positive mode with *m/z* 600-1800 using FlexImaging 5.0 software (Bruker Daltonics). In brief, the acquisition method was calibrated using polyalanine (PolyAla) calibration standard and quadratic calibration. 200 laser shots at 10 kHz repetition speed were accumulated for each raster spot.

Peptide MSI. Prior to peptide MSI, tissue sections were delipidated using the washing procedure by Yang and Caprioli [17]: 70% ethanol (30 s), 100% ethanol (30 s), Carnoy's fluid (60/30/10 ethanol/chloroform/acetic acid v/v/v) (120 s), 100% ethanol (30 s), ddH2O (30 s) and 100% ethanol (30 s).

Peptide MSI was done on a Rapiflex MALDI-TOF MS (Bruker Daltonics) in positive linear mode with *m/z* 2,000-10,000 using FlexImaging 5.0 software (Bruker Daltonics) as described elsewhere [7].

MSI features describing the distribution of several occurrences of β-Amyloids in a peptide dataset of the same specimen, which are related to chosen APP NL-G-F mouse model and the Alzheimer disease, are described in a separate publication by Enzlein et al. [7].

Use-case 1: N-linked glycan *m/z* candidate detection

We re-analyzed a publicly available N-linked glycan MALDI-TOF dataset [34, 42] to demonstrate the applicability of M^2aia. A similar study was done by Föll et al. [29] using the application framework Galaxy that is designed for server-side processing. The dataset is available in the PRIDE repository with accession code PXD009808. The data was published by Gustafsson et al. [34] and used to examine an automated sample preparation approach for MALDI-TOF/TOF imaging of N-linked glycans on formalin-fixed paraffin-embedded (FFPE) murine kidney tissue [34, 42]. PNGase F was printed on two FFPE kidney sections to release N-linked glycans from proteins. A part of the third kidney was covered with N-glycan calibrants and another part with buffer to serve as a control. Imaging was performed with a spatial resolution of 100 µm.

Using M^2aia, we loaded three datasets *(PNG1, PNG2 and control; in total approx. 6.4 GB; skipping the calibrant area)* and applied TIC normalization, Savitzky-Golay smoothing and Top-Hat baseline correction (figure 5, Data preparation). Peak picking with monoisotopic peak identification was applied to the mean spectrum of each image, respectively. The peak results of the datasets were combined into a common peak list. Peak binning was applied to remove duplicates, resulting in a list of 107 *m/z* (candidate) peaks (figure 5, Feature extraction).

To demonstrate how M^2aia can be used in combination with other tools, we exported the processed data as a single imzML file to continue the processing with Cardinal (2.6.0) [43]. Providing the list of common peak features during the export process allows to store the data in continuous centroid format. Using Cardinal, we compare the two PNGase F treated kidney tissue sections with the control tissue section for the identification of discriminant *m/z* candidates that are potentially related to N-linked glycans. N-linked glycan *m/z* candidates were selected by the supervised spatial shrunken centroids (SSC) algorithm [44, 45] (figure 5, Analysis). Therefore, all pixels were separated into the classes "treated" (for PNG1 and PNG2) or "untreated" (for Control). By mapping the *treated* features selected by the SSC to the original publication of Gustafsson et al.

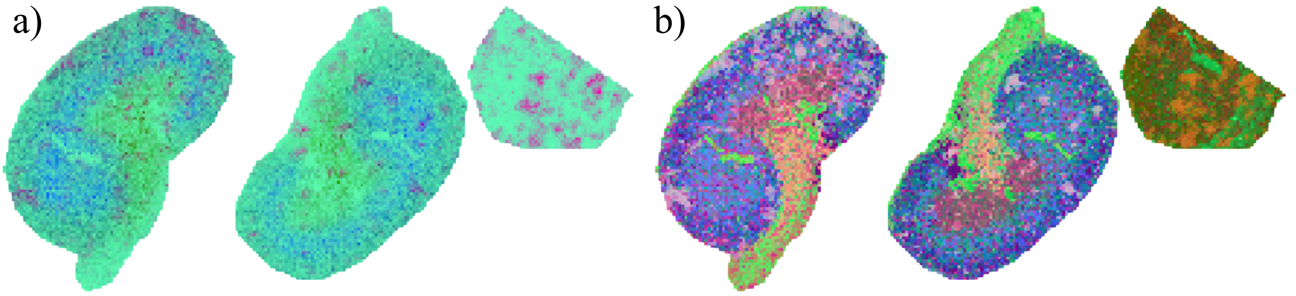

Figure 6. Results of two dimensionality reduction methods after performing the pipeline shown in figure 5. In a) the three principal components with the largest eigenvalues of a PCA and in b) results of a t-SNE with a target dimension of three are shown.

Table 2. Potential *m*/*z*-candidates related to N-linked glycans

Id	*m*/*z*	t-Statistics	Error (ppm)	Error (ppm) Föll et al. [29]	LC-MS/MS M+NA+	Composition
1	1257.4730	63.79	50	51	1257.41	(Hex)$_2$+(Man)$_3$(GlcNAc)$_2$
2	1419.5177	60.62	33	59	1419.47	(Hex)$_3$+(Man)$_3$(GlcNAc)$_2$
3	1743.6281	54.50	33	67	1743.57	(Hex)$_5$+(Man)$_3$(GlcNAc)$_2$
4	1905.6748	50.93	23	30	1905.63	(Hex)$_6$+(Man)$_3$(GlcNAc)$_2$
5	1581.5697	50.58	25	61	1581.53	(Hex)$_4$+(Man)$_3$(GlcNAc)$_2$
6	2304.8962	47.12	28	36	2304.83	(Hex)$_2$(HexNAc)$_3$(deoxyhexose)$_3$+(Man)$_3$(GlcNAc)$_2$
7	1850.7140	46.86	34	34	1850.65	(Hex)$_1$(HexNAc)$_3$(deoxyhexose)$_1$+(Man)$_3$(GlcNAc)$_2$
8	1809.6975	45.44	37	52	1809.63	(Hex)$_2$(HexNAc)$_2$(deoxyhexose)$_1$+(Man)$_3$(GlcNAc)$_2$
9	2158.8425	38.62	33	54	2158.77	(Hex)$_2$(HexNAc)$_3$(deoxyhexose)$_2$+(Man)$_3$(GlcNAc)$_2$
10	1663.6324	35.92	37	58	1663.57	(Hex)$_2$(HexNAc)$_2$+(Man)$_3$(GlcNAc)$_2$
11	1485.5967	33.83	44	63	1485.53	(HexNAc)$_2$(deoxyhexose)$_1$+(Man)$_3$(GlcNAc)$_2$
12	1688.6586	31.59	28	62	1688.61	(HexNAc)$_3$(deoxyhexose)$_1$+(Man)$_3$(GlcNAc)$_2$
13	2012.7717	26.88	30	37	2012.71	(Hex)$_2$(HexNAc)$_3$(deoxyhexose)$_1$+(Man)$_3$(GlcNAc)$_2$
14	1647.6444	26.83	45	-	1647.57	(Hex)$_1$(HexNAc)$_2$(deoxyhexose)$_1$+(Man)$_3$(GlcNAc)$_2$
15	2816.1882	24.31	63	63	2816.01	(Hex)$_3$(HexNAc)$_4$(deoxyhexose)$_1$+(Man)$_3$(GlcNAc)$_2$
16	2067.7292	19.00	28	43	2067.67	(Hex)$_7$+(Man)$_3$(GlcNAc)$_2$

By the re-analysis of the data published by Gustafsson et al. [34] in M^2aia, we were able to identify a set of 16 discriminating *m*/*z* features (col. 2) obtained from the MSI data and mapped it to the LC-MS/MS experiment [34] (col. 6) for the treated kidney sections. Errors are listed for M^2aia (col. 4) and Föll et al. [29] (col. 5) for comparison. Compositions [34] (col. 7) of the corresponding *m*/*z* features. t-Statistics of the supervised spatial shrunken centroids algorithm (col. 3). Identifier (id, col. 1) for sorted features by descending t-statistics. Hex: Hexose, Man: Mannose, GlcNAc: N-Acetyl-D-glucosamine, HexNAc: N-Acetyl-D-hexosamine.

[34], we could identify 16 N-linked glycan related *m*/*z* candidates, as listed in table 2. We calculated PCA images including the first three principal components and a t-SNE image (figure 6) based on the common peak list in M^2aia.

For reproducibility purposes, protocols of the interactive steps from loading to exporting [46] and for dimensionality reduction [47] are available. The R-based processing of the intermediate results is available as a CodeOcean capsule [48, 49]. An additional CodeOcean capsule implements the described workflow as a command-line application [50, 51], demonstrating the possibility to develop M^2aia-based applications for batch-processing and porting them to a server infrastructure.

Use-case 2: Multi-modal 3D image reconstruction

As mentioned before, the dataset published together with this paper consists of 10 consecutive brain slices of a APP NL-G-F mouse model, imaging both lipid and peptide features *(in total approx. 80 GB in size.)* The objective of use-case described in the following is to demonstrate the applicability of M^2aia for mono- and multi-modal 3D image reconstructions by showing how to embed the peptide information into the lipid structural context in three dimensions.

To demonstrate mono-modal 3D reconstruction, all 10 slices of the lipid and the peptide datasets were loaded into M^2aia, respectively, and used for slice-wise reconstruction of 3D image stacks. For multi-modal 3D-reconstruction, the peptide dataset was pair-wise registered with the respective lipid slices of the previously reconstructed 3D lipid image stack.

Each of the 10 lipid imzML binary files is about 4.9 GB on disk and each of the 10 peptide imzML binary files is about 2.8 GB. Loading and initialization of a single lipid image into M^2aia took 3.26 $\pm$ 0.67 seconds for the lipid data and 2.06 $\pm$ 0.45 seconds for the peptide data. During the initialization, maps of TIC normalization factors and the TIC normalized overview spectra are created for each dataset.

Successful image-based registration requires images that are rich and similar in structural features. This can be done in M^2aia by fast and interactive exploration of ion images. For the example data, structure-rich images in the lipid dataset were found at *m*/*z* 865$\pm$0.65 Da and for the peptide dataset at *m*/*z* 2250$\pm$50 Da. For a rough initial alignment of considerably rotated tissue sections, we took advantage of M^2aia's capability to interactively rotate the slices by $\pm$15 degrees around the center. Additionally, the non-tissue areas were removed from the ion image generation process by segmentation of the respective areas, using the segmentation tools provided by M^2aia.

For mono-modal 3D reconstructions, a reference slice was first selected in the corresponding M^2aia plugin from the list of ordered slices. Starting from this reference slice, adjacent slices were automatically aligned to each other by rigid and deformable image-based registration. The process is applied to the image stack in both downward and upward directions (see 3D reconstruction section of figure 7).

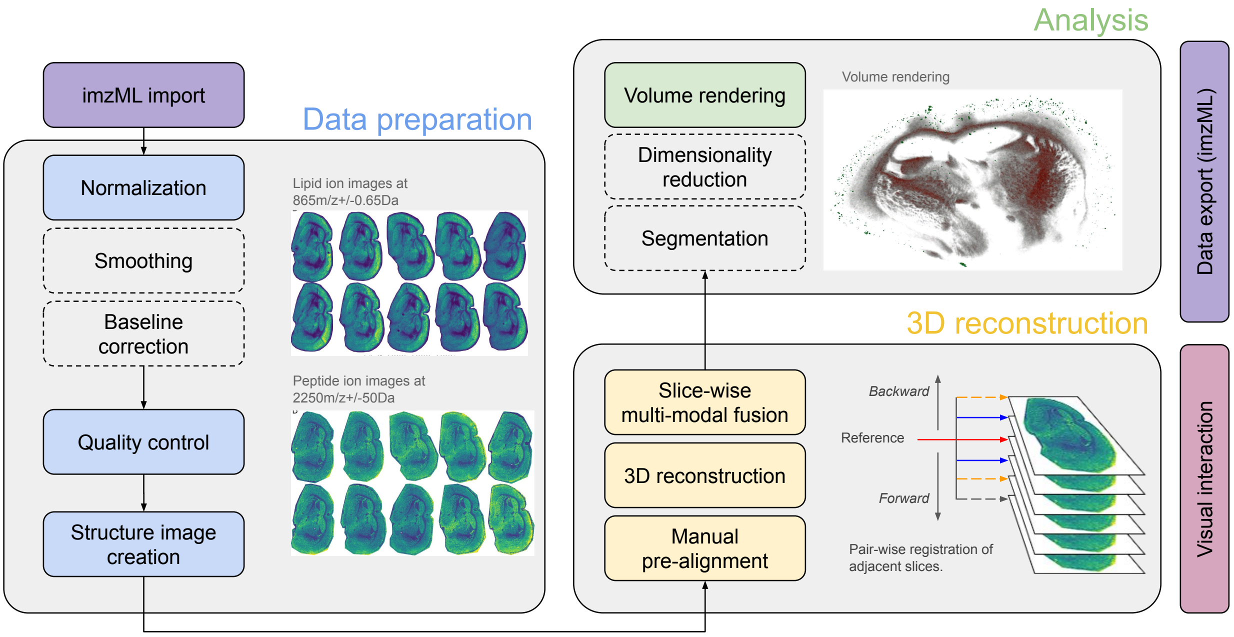

Figure 7. Steps for 3D reconstruction of consecutive MSI image slices in M^2aia, exemplified by images from the 3D reconstruction and *registration* of the publicly available MALDI-TOF lipid and peptide dataset. Dashed boxes are possible additional processing steps that were not applied to the data shown.

Rigid registration is based on a multi-resolution registration strategy (Gaussian pyramid with three levels and down-sampling factors of 4,2,1). Advanced Mattes Mutual Information [24] is used as metric for the optimization of a Euler transformation using linear interpolation and 250 iterations.

For the subsequent deformable registration steps, the same multi-resolution scheme and metric are applied. As deformable transformation, a recursive B-Spline transformation is used with final grid spacing on the original resolution set to 0.8mm, with scaling factors per pyramid level of 2, 1.5 and 1, respectively. Interpolation is performed by third-order B-Splines. The optimization is run for 750 iterations. Figure 7 summarizes the workflow.

To quantify the accuracy of the registration, we used M^2aia to interactively select seven reference points in each slice and in both modalities independently (a subset of points share a common anatomical location in both modalities), resulting in 70 reference points per set. In figure 8 the reference points of both modalities are shown in context of the reconstructed lipid dataset for the mid-slice of the stack.

For the mono-modal 3D reconstructions of the lipid (see figure 4) and peptide datasets, we obtained a mean registration error of 28±8 µm and 35±5 µm, respectively, and for the multi-modal reconstruction a mean reconstruction error of 39 ± 4 µm.

A protocol [52] showing how to perform the interactive steps in M^2aia of the workflow as described above is available on protocols.io. Additional file 1 shows a rotating view of the volume visualization of the reconstructed data.

Discussion

M^2aia's multi-threading and lazy loading concepts enable memory efficient exploration of datasets that are far larger than the system's actual working memory (Figure 2*)*. As shown in Table 1, loading a 44.2 GB dataset requires less than 500 MB of RAM. This allows even complex MSI analysis tasks to be performed on standard PCs. We demonstrate this for m/z candidate detection on an N-linked glycan MALDI-TOF dataset and 3D multi-modal *registration* of a lipid and peptide dataset. All steps of the use-cases were performed on a laptop computer with 16 GB of RAM.

The graphical user interface (GUI) of M^2aia is intended to remove existing barriers related to performing the steps of an MSI study. Additionally, all features of the M^2aia programming API can also be used to create commandline applications. This is beneficial for batch processing of huge databases of MS images. In this case the interactive M^2aia GUI can help to understand the implications of each processing step during development and quality control.

In addition to interactive processing with M^2aia, it is possible to create memory-efficient command-line applications that also benefit from the multi-threading and lazy loading approaches. By implementing command-line applications within an OS-level virtualization (e.g. Docker) a scalable, distributable, and reproducible server-side MSI processing environment can be created and integrated with established workflow tools such as Galaxy.

Image-to-image registration requires structure-rich images that include common characteristic features between tis-

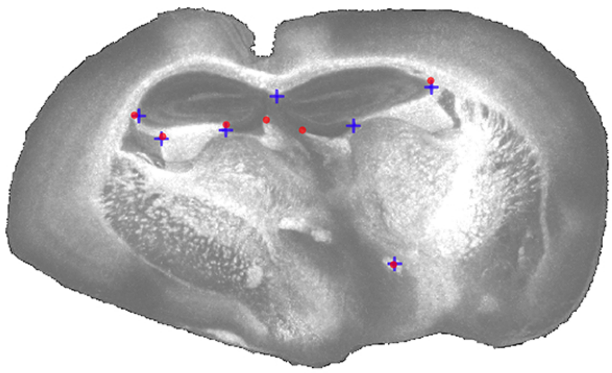

Figure 8. Maximum intensity projection of the multi-modal 3D reconstructed dataset at m/z 865±0.65 Da. Peptide (blue crosses) and lipid (red circles) reference points are shown for the mid-slice of the stack.

sue slices or, for the multi-modal case, between modalities. This may require a user-driven search for structure-rich images across the *m/z* dimension, which is facilitated by the fast and interactive ion image generation of M^2*aia*. Since different masses are intrinsically registered, it is irrelevant whether the structures visible in an image are meaningful entities or imaging/normalization artefacts. To demonstrate this, an unusually wide mass range of 50 Da at *m/z* 2250 was chosen to generate a structure-rich image. This was successfully used in use-case 2 for the 3D reconstruction. Disabling TIC normalization for the same *m/z*-range leads to a noisy image without structures, not usable for image-based registration - suggesting that the contrast is actually caused by a TIC normalization artefact.

Another challenge for purely automatic image registration approaches are significantly misaligned, especially heavily rotated or, even worse, flipped images. The interactive environment of M^2aia allows to quickly obtain a rough pre-alignment of the images that is sufficient as initialization for subsequent automatic refinement. With the possibility to edit the elastix parameter file, M^2aia offers unrestricted access to the full potential of the elastix toolkit to enable problem-specific customization of image registration.

Evaluation of registration results is yet another task that requires interaction. Either it is performed qualitatively by visualization methods (like blending or checkerboard visualization), or quantitatively by comparing corresponding landmarks or, less accurately, segmentations. Both typically require interactive tools, e.g. to select the appropriate parameters for visualization, to define corresponding landmarks or to perform (or at least verify) segmentations. MITK, the toolkit M^2aia is based on, offers such tools. Future releases of M^2aia will make them more easily accessible for MSI data.

With rare exceptions, transforming an image to another coordinate system requires interpolation of image data. If interpolation is applied to spectral data, the interpolated spectra must be interpreted with caution. To avoid possible misinterpretation of interpolated spectra, M^2aia currently calculates only interpolated ion images and allows to store the transformation parameters for use together with the unmodified MSI data. To avoid interpolation of spectra in a multi-modal registration task with MSI and non-MSI data, the MSI image domain should be used as the fixed image domain.

Multi-modal MS imaging refers to approaches with different MSI contrasts (like lipid and peptide MS imaging) as well as combined MSI and non-MS imaging methods, e.g. MSI combined with microscopy. M^2aia's capabilities for the former scenario were demonstrated in use case 2. Combining MSI and microscopy is a common multi-modal MSI experiment with its own challenges in interactive visualization. Due to the high lateral resolution of microscopy images, memory-efficient handling of microscopy data sets requires pyramidal and tiled storage approaches. To enable this in M^2aia we are currently developing an interface for reading whole-slide images by utilizing the OpenSlide library [53]. This will be part of an upcoming release of M^2aia.

Conclusion

To our knowledge, M^2*aia* is the first open-source application that provides interactive, fast and highly memory efficient access to multiple 2D/3D MS images at the same time. It offers all steps of a typical MSI signal processing work flow, responsive visual interaction in 2D and 3D as well as image processing functions like segmentation and features deformable 3D reconstruction and multi-modal *registration*. Another unique feature is that fused data with individual mass axes can be visualized in a shared coordinate system. Furthermore, M^2*aia* is an extensible framework allowing the development of custom MSI analysis pipelines.

Additional Information

- Additional file 1 (.mp4) - Volume visualization of the multi-modal 3D reconstruction. A rotating view was generated for the result of use-case 2, showing a volume visualization of *m/z* 864±0.65 Da in blue-violet and *m/z* 4070±5 Da in green.

Availability of Data and Material

- M^2*aia* binaries for Windows and Linux [54].
- M^2*aia* source code [55]
- The 3D reference datasets by Oetjen et al. [5] used to generate the results of the timing experiments are available on the MetaboLights repository with accession code MTBLS176.
- Supporting data of use-case 1: N-linked glycan MALDI MSI datasets by Gustafsson et al. [42] is available in the PRIDE repository with accession code PXD009808.
- Supporting protocol of use-case 1: interactive steps from loading to exporting [46].
- Supporting protocol of use-case 1: dimensionality reduction [46].
- Supporting capsule of use-case 1: command-line application based processing [50].
- Supporting capsule of use-case 1: R-based processing [48].
- Supporting data of use-case 2: multi-modal 3D lipid and peptide mouse brain MSI data data have been deposited to the GigaDB repository [36].
- Supporting protocol of use-case 2: interactive steps of 3D image reconstruction [52].

Availability of Supporting Source Code and Requirements

- Project name: M^2*aia*, RRID:SCR_019324
- Project home page: Github repository [56]
- Operating systems: Windows and Unix
- Programming language: C++, CMake and R
- Other requirements: The project is based on the MITK snapshot/2020-12-21 [57] (required for compiling) and elastix (RRID:SCR_009619) v5.0.0 binaries [58] (required for running)
- License: BSD

Ethics, consent and permissions

All experiments were approved (No.142/2015) by the Ethics Committee on Animal Experimentation of the University of Leuven.

Abbreviations

CPU: Central Processing Unit; DB: database; DR: dimensionality reduction; GB: Gigabyte; H&E: hematoxylin-eosin; ibd: imaging binary data; imzML: imaging mz Markup Language; ITK: Insight Toolkit; LC-MS/MS: liquid chromatography tandem mass spectrometry; JIP: Joint Imaging Platform; MB: Megabyte; M^2*aia*: MSI applications for interactive analysis in MITK; MALDI: matrix-assisted laser desorption/ionization; MS: mass spectrometry; MSI: mass spectrometry imaging; MITK: Medical Imaging Interaction Toolkit; mz or *m/z*: mass-to-charge ratio; PCA: pricipal components analysis; ppm: parts per million; PRIDE: proteomics identifica-

tions; SSC: spatial shrunken centroids; SSD: Solid-State-Drive; t-SNE: t-Stochastic Neighborhood Embedding; TIC: total ion count/current; TOF: time of flight;

Competing Interests

The authors declare that they have no competing interests.

Funding

This work was funded by the German Federal Ministry of Research (BMBF) as part of the Innovation Partnership M^2*Aind*, project M^2*OGA* (13FH8I02IA) within the framework FH-Impuls.

Authors' Contributions

J.C. developed and tested M^2*aia*, created the use-cases and protocols. T.E. and C.M. prepared and acquired the lipid and peptide 3D data that were made publicly available. T.E. and M.H. helped testing the application. M.H. contributed to the development of the visualization strategies. I.W. and C.H. contributed to the conceptualization and funding acquisition. J.C. and I.W. wrote the manuscript. All authors critically read and approved the manuscript's contents.

Acknowledgments

We thank the MITK developer team for the provisioning and continuous maintenance of the application backbone. M^2*aia*'s t-SNE feature includes software developed by the Delft University of Technology.

References

1. McDonnell LA, Römpp A, Balluff B, Heeren RMA, Albar JP, Andrén PE, et al. Discussion point: reporting guidelines for mass spectrometry imaging. Analytical and Bioanalytical Chemistry. 2015 Mar. http://dx.doi.org/10.1007/s00216-014-8322-6.
2. Schulz S, Becker M, Groseclose MR, Schadt S, Hopf C. Advanced MALDI mass spectrometry imaging in pharmaceutical research and drug development. Current Opinion in Biotechnology. 2019 Feb. http://dx.doi.org/10.1016/j.copbio.2018.08.003.
3. Paine MRL, Liu J, Huang D, Ellis SR, Trede D, Kobarg JH, et al. Three-Dimensional Mass Spectrometry Imaging Identifies Lipid Markers of Medulloblastoma Metastasis. Scientific Reports. 2019 Dec. http://dx.doi.org/10.1038/s41598-018-38257-0.
4. Buchberger AR, DeLaney K, Johnson J, Li L. Mass Spectrometry Imaging: A Review of Emerging Advancements and Future Insights. Analytical Chemistry. 2018 Jan. http://dx.doi.org/10.1021/acs.analchem.7b04733.
5. Oetjen J, Veselkov K, Watrous J, McKenzie JS, Becker M, Hauberg-Lotte L, et al. Benchmark datasets for 3D MALDI- and DESI-imaging mass spectrometry. GigaScience. 2015 Dec. http://dx.doi.org/10.1186/s13742-015-0059-4.
6. Ràfols P, Vilalta D, Brezmes J, Cañellas N, del Castillo E, Yanes O, et al. Signal preprocessing, multivariate analysis and software tools for MA(LDI)-TOF mass spectrometry imaging for biological applications: MSI DATA PROCESSING. Mass Spectrometry Reviews. 2018 May. http://dx.doi.org/10.1002/mas.21527.
7. Enzlein T, Cordes J, Munteanu B, Michno W, Serneels L, De Strooper B, et al. Computational Analysis of Alzheimer Amyloid Plaque Composition in 2D- and Elastically Reconstructed 3D-MALDI MS Images. Analytical Chemistry. 2020 Oct. http://dx.doi.org/10.1021/acs.analchem.0c02585.
8. Palmer AD, Alexandrov T. Serial 3D Imaging Mass Spectrometry at Its Tipping Point. Analytical Chemistry. 2015 Apr. http://dx.doi.org/10.1021/ac504604g.
9. Thiele H, Heldmann S, Trede D, Strehlow J, Wirtz S, Dreher W, et al. 2D and 3D MALDI-imaging: Conceptual strategies for visualization and data mining. Biochimica et Biophysica Acta (BBA) - Proteins and Proteomics. 2014 Jan. http://dx.doi.org/10.1016/j.bbapap.2013.01.040.
10. Trede D, Schiffler S, Becker M, Wirtz S, Steinhorst K, Strehlow J, et al. Exploring Three-Dimensional Matrix-Assisted Laser Desorption/Ionization Imaging Mass Spectrometry Data: Three-Dimensional Spatial Segmentation of Mouse Kidney. Analytical Chemistry. 2012 Jul. http://dx.doi.org/10.1021/ac300673y.
11. Patterson NH, Doonan RJ, Daskalopoulou SS, Dufresne M, Lenglet S, Montecucco F, et al. Three-dimensional imaging MS of lipids in atherosclerotic plaques: Open-source methods for reconstruction and analysis. PROTEOMICS. 2016 Jun. http://dx.doi.org/10.1002/pmic.201500490.
12. Abdelmoula WM, Regan MS, Lopez BGC, Randall EC, Lawler S, Mladek AC, et al. Automatic 3D Nonlinear Registration of Mass Spectrometry Imaging and Magnetic Resonance Imaging Data. Analytical Chemistry. 2019 May. http://dx.doi.org/10.1021/acs.analchem.9b00854.
13. Hanrieder J, Phan NTN, Kurczy ME, Ewing AG. Imaging Mass Spectrometry in Neuroscience. ACS Chemical Neuroscience. 2013 May. http://dx.doi.org/10.1021/cn400053c.
14. Kaya I, Brinet D, Michno W, Başkurt M, Zetterberg H, Blenow K, et al. Novel Trimodal MALDI Imaging Mass Spectrometry (IMS3) at 10 µm Reveals Spatial Lipid and Peptide Correlates Implicated in Aβ Plaque Pathology in Alzheimer's Disease. ACS Chemical Neuroscience. 2017 Dec. http://dx.doi.org/10.1021/acschemneuro.7b00314.
15. Kaya I, Zetterberg H, Blennow K, Hanrieder J. Shedding Light on the Molecular Pathology of Amyloid Plaques in Transgenic Alzheimer's Disease Mice Using Multimodal MALDI Imaging Mass Spectrometry. ACS Chemical Neuroscience. 2018 Jul. http://dx.doi.org/10.1021/acschemneuro.8b00121.
16. Kaya I, Sämfors S, Levin M, Borén J, Fletcher JS. Multimodal MALDI Imaging Mass Spectrometry Reveals Spatially Correlated Lipid and Protein Changes in Mouse Heart with Acute Myocardial Infarction. Journal of the American Society for Mass Spectrometry. 2020 Oct. http://dx.doi.org/10.1021/jasms.0c00245.
17. Yang J, Caprioli RM. Matrix Sublimation/Recrystallization for Imaging Proteins by Mass Spectrometry at High Spatial Resolution. Analytical Chemistry. 2011 Jul. http://dx.doi.org/10.1021/ac200998a.
18. Abdelmoula WM, Škrášková K, Balluff B, Carreira RJ, Tolner EA, Lelieveldt BPF, et al. Automatic Generic Registration of Mass Spectrometry Imaging Data to Histology Using Nonlinear Stochastic Embedding. Analytical Chemistry. 2014 Sep. http://dx.doi.org/10.1021/ac502170f.
19. Patterson NH, Tuck M, Van de Plas R, Caprioli RM. Advanced Registration and Analysis of MALDI Imaging Mass Spectrometry Measurements through Autofluorescence Microscopy. Analytical Chemistry. 2018 Nov. http://dx.doi.org/10.1021/acs.analchem.8b02884.
20. Van de Plas R, Yang J, Spraggins J, Caprioli RM. Image fusion of mass spectrometry and microscopy: a multimodality paradigm for molecular tissue mapping. Nature Methods. 2015 Apr. http://dx.doi.org/10.1038/nmeth.3296.

21. Verbeeck N, Yang J, De Moor B, Caprioli RM, Waelkens E, Van de Plas R. Automated Anatomical Interpretation of Ion Distributions in Tissue: Linking Imaging Mass Spectrometry to Curated Atlases. Analytical Chemistry. 2014 Sep. http://dx.doi.org/10.1021/ac502838t.
22. McCormick M, Liu X, Jomier J, Marion C, Ibanez L. ITK: enabling reproducible research and open science. Frontiers in Neuroinformatics. 2014. http://dx.doi.org/10.3389/fninf.2014.00013.
23. Insight Toolkit (ITK), `http://www.itk.org`. Accessed 12 Jan. 2021.
24. Klein S, Staring M, Murphy K, Viergever MA, Pluim J. elastix: A Toolbox for Intensity-Based Medical Image Registration. IEEE Transactions on Medical Imaging. 2010 Jan. http://dx.doi.org/10.1109/TMI.2009.2035616.
25. Abdelmoula WM, Pezzotti N, Hölt T, Dijkstra J, Vilanova A, McDonnell LA, et al. Interactive Visual Exploration of 3D Mass Spectrometry Imaging Data Using Hierarchical Stochastic Neighbor Embedding Reveals Spatiomolecular Structures at Full Data Resolution. Journal of Proteome Research. 2018 Mar. http://dx.doi.org/10.1021/acs.jproteome.7b00725.
26. Nolden M, Zelzer S, Seitel A, Wald D, Müller M, Franz AM, et al. The Medical Imaging Interaction Toolkit: challenges and advances : 10 years of open-source development. International Journal of Computer Assisted Radiology and Surgery. 2013 Jul. http://dx.doi.org/10.1007/s11548-013-0840-8.
27. Schramm T, Hester Z, Klinkert I, Both JP, Heeren RMA, Brunelle A, et al. imzML — A common data format for the flexible exchange and processing of mass spectrometry imaging data. Journal of Proteomics. 2012 Aug. http://dx.doi.org/10.1016/j.jprot.2012.07.026.
28. Afgan E, Baker D, Batut B, van den Beek M, Bouvier D, Čech M, et al. The Galaxy platform for accessible, reproducible and collaborative biomedical analyses: 2018 update. Nucleic Acids Research. 2018 Jul. http://dx.doi.org/10.1093/nar/gky379.
29. Föll MC, Moritz L, Wollmann T, Stillger MN, Vockert N, Werner M, et al. Accessible and reproducible mass spectrometry imaging data analysis in Galaxy. GigaScience. 2019 Dec. http://dx.doi.org/10.1093/gigascience/giz143.
30. Schroeder W, Martin K, Lorensen B. The visualization toolkit: an object-oriented approach to 3D graphics. 4. ed ed. Clifton Park, NY: Kitware, Inc; 2006. OCLC: 255911428.
31. Visualization Toolkit (VTK), `http://www.vtk.org`. Accessed 12 Jan. 2021.
32. The Qt Company, `http://www.qt.io`. Accessed 12 Jan. 2021.
33. Scherer J, Nolden M, Kleesiek J, Metzger J, Kades K, Schneider V, et al. Joint Imaging Platform for Federated Clinical Data Analytics. JCO Clinical Cancer Informatics. 2020 Nov. http://dx.doi.org/10.1200/CCI.20.00045.
34. Gustafsson OJR, Briggs MT, Condina MR, Winderbaum LJ, Pelzing M, McColl SR, et al. MALDI imaging mass spectrometry of N-linked glycans on formalin-fixed paraffin-embedded murine kidney. Analytical and Bioanalytical Chemistry. 2015 Mar. http://dx.doi.org/10.1007/s00216-014-8293-7.
35. Perez-Riverol Y, Csordas A, Bai J, Bernal-Llinares M, Hewapathirana S, Kundu DJ, et al. The PRIDE database and related tools and resources in 2019: improving support for quantification data. Nucleic Acids Research. 2019 Jan. http://dx.doi.org/10.1093/nar/gky1106.
36. Cordes J, Enzlein T, Marsching C, Hinze M, Engelhardt S, Hopf C, et al. Supporting data for "*M^2aia* - Interactive fast and memory efficient analysis of 2D and 3D multi-modal mass spectrometry imaging data". GigaScience Database. 2021 Jan. http://dx.doi.org/PENDING.
37. van Herk M. A fast algorithm for local minimum and maximum filters on rectangular and octagonal kernels. Pattern Recognition Letters. 1992 Jul. http://dx.doi.org/10.1016/0167-8655(92)90069-C.
38. Savitzky A, Golay MJE. Smoothing and Differentiation of Data by Simplified Least Squares Procedures. Analytical Chemistry. 1964 Jul. http://dx.doi.org/10.1021/ac60214a047.
39. Breen E, Hopewood F, Williams K, Wilkins M. Automatic Poisson peak harvesting for high throughput protein identification. ELECTROPHORESIS ER. 2000 Jun. http://dx.doi.org/10.1002/1522-2683(20000601)21:11<2243::AID-ELPS2243>3.0.CO;2-K.
40. Van der Maaten L, Courville A, Fergus R, Manning C. Accelerating t-SNE using Tree-Based Algorithms. Journal of Machine Learning Research. 2014 Oct;15:3221–3245.
41. Nilsson P, Saito T, Saido TC. New Mouse Model of Alzheimer's. ACS Chemical Neuroscience. 2014 Jul. http://dx.doi.org/10.1021/cn500105p.
42. Gustafsson OJR, Briggs MT, Condina MR, Winderbaum LJ, Pelzing M, McColl SR, et al. Raw N-glycan mass spectrometry imaging data on formalin-fixed mouse kidney. Data in Brief. 2018 Dec. http://dx.doi.org/10.1016/j.dib.2018.08.186.
43. Bemis KD, Harry A, Eberlin LS, Ferreira C, van de Ven SM, Mallick P, et al. Cardinal: an R package for statistical analysis of mass spectrometry-based imaging experiments. Bioinformatics. 2015 Jul. http://dx.doi.org/10.1093/bioinformatics/btv146.
44. Tibshirani R, Hastie T, Narasimhan B, Chu G. Class Prediction by Nearest Shrunken Centroids, with Applications to DNA Microarrays. Statistical Science. 2003 Feb. http://dx.doi.org/10.1214/ss/1056397488.
45. Bemis KD, Harry A, Eberlin LS, Ferreira CR, van de Ven SM, Mallick P, et al. Probabilistic Segmentation of Mass Spectrometry (MS) Images Helps Select Important Ions and Characterize Confidence in the Resulting Segments. Molecular & Cellular Proteomics. 2016 May. http://dx.doi.org/10.1074/mcp.O115.053918.
46. Cordes J. Supporting protocol for use-case 1: N-linked glycan m/z candidate detection in "M2aia - Interactive, fast and memory efficient analysis of 2D and 3D multi-modal mass spectrometry imaging data". protocols.io. 2021 Jan. http://dx.doi.org/10.17504/protocols.io.brw2m7ge.
47. Cordes J. Supporting protocol for use-case 1: Dimensionality reduction in "M2aia - Interactive, fast and memory efficient analysis of 2D and 3D multi-modal mass spectrometry imaging data". protocols.io. 2021 Jan. http://dx.doi.org/PENDING.
48. Cordes J. Supporting capsule for use-case 1: R-based processing in "M^{2}aia - Interactive, fast and memory efficient analysis of 2D and 3D multi-modal mass spectrometry imaging data". CodeOcean. 2021 Jan. http://dx.doi.org/PENDING.
49. *M^2aia* Github repository; R-based processing for use-case 1 "N-linked glycan m/z candidate detection", `https://github.com/jtfcordes/m2aia/blob/v2021.01.01/Scripts/N-Glycan-CandidateDetection_UseCase1.R`. Accessed 27 Jan. 2021.
50. Cordes J. Supporting capsule for use-case 1: Command-line application based pre-processing in "M^{2}aia - Interactive, fast and memory efficient analysis of 2D and 3D multi-modal mass spectrometry imaging data". CodeOcean. 2021 Jan. http://dx.doi.org/PENDING.
51. *M^2aia* Github repository; Command-line application for use-case 1 "N-linked glycan m/z candidate detection", `https://github.com/jtfcordes/m2aia/blob/v2021.01.01/Modules/M2aiaCLI/NLinkedGlycan.cpp`. Accessed 27 Jan.

2021.
52. Cordes J. Supporting protocol for use-case 2: Multi-modal 3D image reconstruction in "M2aia - Interactive, fast and memory efficient analysis of 2D and 3D multi-modal mass spectrometry imaging data". protocols.io. 2021 Jan. http://dx.doi.org/PENDING.
53. Satyanarayanan M, Goode A, Gilbert B, Harkes J, Jukic D. OpenSlide: A vendor-neutral software foundation for digital pathology. Journal of Pathology Informatics. 2013. http://dx.doi.org/10.4103/2153-3539.119005.
54. M^2aia; Github Realese Page 2021.01.01, https://github.com/jtfcordes/m2aia/releases/tag/v2021.01.01. Accessed 27 Jan. 2021.
55. M^2aia; Archived Release version 2021.01.01, https://doi.org/10.5281/zenodo.4471358. Accessed 27 Jan. 2021.
56. M^2aia; Github repository, https://www.github.com/jtfcordes/m2aia. Accessed 27 Jan. 2021.
57. MITK Github repository; snapshots/2020-12-21, https://github.com/MITK/MITK/tree/snapshots/2020-12-21. Accessed 12 Jan. 2021.
58. elastix image registration toolbox Github repository; v5.0.0, https://github.com/SuperElastix/elastix/releases/tag/5.0.0. Accessed 19 Jan. 2021.

Additional file 1(.mp4) Volume visualization of the multi-modal 3D reconstruction

Click here to access/download

Supplementary Material

AdditionalFile_1.mp4
