# Supplementary material for: M2aia—Interactive, fast, and memory-efficient analysis of 2D and 3D multi-modal mass spectrometry imaging data
Source: Gigascience. 2021 Jul 20;10(7):giab049. doi: 10.1093/gigascience/giab049 (PMC8290197; doi:10.1093/gigascience/giab049)
Supplement: giab049_Response_to_Reviewer_Comments_Original_Submission [file giab049_response_to_reviewer_comments_original_submission.pdf]

Author's Response To Reviewer Comments

Close

We sincerely thank the reviewers for their thoughtful comments and suggestions. We have improved the manuscript by carefully addressing the issues raised and provide a point-by-point response below. In the manuscript, we have highlighted major changes in blue to allow an easier tracking of the changes.

Reviewer #1: This excellent Technical Note describes M2aia (MSI applications for interactive analysis in MITK), a software tool enabling interactive signal processing and visualisation of Mass spectrometry imaging (MSI) datasets. As the authors explain, M2aia extends the well used Medical Imaging and Interaction Toolkit (MITK) (Nolden et al., Journal of Computer Assisted Radiology and Surgery. 2013 Jul.). Key features of the M2aia toolkit include image segmentation, dimensionality reduction, and elastix-based image registration. Importantly, M2aia supports read/write access to the open standard imaging format mz Markup Language (imzML) that is used for MSI data.

The authors highlight the use of M2aia through two use cases. The first use case explored N-linked glycan m/z candidate detection in formalin-fixed paraffin-embedded (FFPE) murine kidney tissue. The latter 3D reference dataset has previously been published in GigaScience (Oetjen et al., GigaScience, Volume 4, Issue 1, December 2015, s13742-015-0059-4), and the authors used this reference dataset to showcase dimensionality reduction and R-based processing of MSI data. The second use case was a study of multi-modal 3D image reconstruction in 10 consecutive brain slices, and this and was used to determine the spatial distribution of brain lipids and brain peptides in an APP NL-G-F mouse model. Importantly, the supporting APP NL-G-F mouse brain lipid and protein MALDI MSI data have been submitted to the GigaScience DataBase (GigaDB), as have the intermediate results of the N-glycan MALDI mouse kidney use case study.

The M2aia software tool is made publicly available on GitHub (https://github.com/jtfcordes/m2aia) where it has been ascribed an OSI-approved BSD-3-Clause License.I recommend this manuscript for publication in GigaScience.

>> Authors:
We thank the reviewer for highlighting the technical aspects in his summary of our work and appreciate the review.

Reviewer #2: The M2aia manuscript describes a set of routines that enable fast and memory efficient analysis of 2D and 3D multimodal mass spectrometry imaging datasets. The routines consist of data input, routine mass spectral processing of [time-of-flight-based] MALDI mass spectrometry MSI data, coregistration with histology or between MSI datasets, generation and visualisation of 3D MSI datasets, and basic dimensionality reduction methods (PCA and tSNE). From the perspective of MSI itself few of the routines are innovative in their own right, for instance the data reduction methods described here parallel those this reviewer published previously [J Am Soc Mass Spectrom. 2010 21:1969-78] and which have been adopted and refined by other groups since. Similarly the registration methods are also similar to those reported previously and which are correctly cited in the current manuscript. However many of those routines have remained academic exercises that have, much to this reviewer's chagrin, not been adequately disseminated to the wider community. The principal advantage of the work is that it provides powerful open source methods that the MSI community can adopt, exploit and improve further. So while it may be lamented that the routines seem overly focused on time-of-flight based MALDI MSI this reviewer would expect them to work very well for FTICR and Orbitrap data; similarly it may be correct that the choices for mass spectral processing or data reduction are rather limited in scope, and do not represent all of the tools that have been previously used, however that would be unfair. The authors have made an open source set of routines that enable multimodal 2D and 3D MSI, and which can be supplemented with additional routines/tools. It is a commendable effort that this reviewer is more than willing to recommend is published in its current status.

>> Authors:
We thank the reviewer for his thoughtful feedback and conclusions.

Reviewer #3: The authors present an interesting software tool for interactive visualization and analysis of mass spectrometry imaging data and its integration with other imaging modalities. The paper is well presented and the authors' generosity to make it publicly available will be highly appreciated by the community. I very much appreciate the development of this tool, and below are a few suggestions that would be great to address:

>> Reviewer #3 – comment 1:
1- The authors mentioned "memory efficient" analysis, but it was not clear how they implement the memory efficient algorithm. Figure 2 would need some clarification. The results show data from two case studies with relatively smaller file size (data size of one case was reported 4.9 and 2.8 GB but the file size of the other case was not reported, please add). While in Table 1, the authors referred to much larger size data, how did they analyze 44.2GB of 3D mouse kidney data with only 235.1MB RAM usage? How is peak picking efficiently performed uploading full data? Please clarify this interesting point, especially since you highlight this as a problem in the field with continuous increase in data size.

>> Authors:
We thank the reviewer for pointing out that Figure 2 did not sufficiently clarify the implementation of the memory-efficient algorithms. Therefore, we have completely reworked Figure 2 and added new elements that highlight the data flow in memory-efficient processing tasks by utilizing multi-threading and lazy-loading. We also updated the caption to clarify the newly added aspects.

We added file size information to use-case 1. We want to clarify at this point (and tried to further emphasize this in the text), that in use-case 2 each single lipid and each single peptide image has a file size of about 4.9 GB and about 2.8 GB, respectively. The whole multi-modal 3D reconstruction process needs to process all ten lipid and all ten peptide files to M²aia, accumulating to an overall data processing volume of about 80 GB.

To clarify memory usage, we added equation (1) to the Data Handling section (on page 4) that describes memory utilization during processing of a single MS image representation in M²aia. We renamed some symbols to more consistently match the text with the content of figure 2: Set of spectra S and images I is now \hat{S} and \hat{I}, respectively. We added pixel type information to overview spectra and images.

Regarding that according to Table 1 only 435.1MB RAM was necessary to analyze 44.2GB of 3D mouse kidney data: 435.1MB RAM was sufficient, because for processing it is not necessary to load all spectral information, but only \hat{S} and \hat{I}. In this case, elements of \hat{I} are 3D images without full spectrum data, but offering memory-side single-ion, normalization, mask, and spectrum-index image data.

To show how "memory efficient" processing performs on current desktop system configurations and the largest reference dataset, we have added new timing experiment results to Table 1.

Regarding peak picking and other pixel-wise processing: This can be performed in a largely file-size-independent and memory efficient manner - as shown in the reworked Figure 2 as Data Processing Task (5) - by means of the multi-threading and lazy loading approaches.

We thank the reviewer for pointing out these important-to-clarify aspects.

>> Reviewer #3 – comment 2:
2- In the discussion, it would be great to discuss how you would envision the future development of this tool in light of analyzing big MSI data? For instance, if I have an imzML file of 100 GB or more, can I analyze it using the current version of this tool (how much memory would be needed?) or are you going to further extend it up for more scalable level?

>> Authors:
Regarding future developments, we have added remarks referring to our current developments to the last paragraph of the Discussion section on page 10.

Regarding the processing of imzML files of 100 GB or more: As we try to clarify above in our response to "Reviewer #3 - comment 1", the size of the data processed for use-case 2 was approx. 80 GB and thus in the order of magnitude of a dataset of 100 GB. For the calculation of how much memory is needed,

please see also the response to "Reviewer #2 - comment 1".

>> Reviewer #3 – comment 3:
3- Image registration: there have been great efforts already done to automate the deformable registration between mass spectrometry imaging and other modalities. The authors covered key papers in the bibliography, but it would be valuable to shed more light either in the introduction or discussion to highlight the challenges, how the published methods addressed the challenges and how your tool benefited from it (if it did), and what are the existing challenges that still need to be addressed in this active research area.

>> Authors:
We added a more detailed discussion of image registration to the Discussion section on page 10.

Reviewer #4: The authors describe their software for MSI visualization and interactive analysis that is built on an existing image visualization and analysis framework, integrating the strength and development of the medical imaging community with MSI data.

Overall, this appears to be an excellent work and responds to the need and desires of the MSI community. While there have been multiple publications on 3D MSI, an open-source tool with a GUI for human-in-the-loop preprocessing and setup has yet to emerge and the described software can meet that need and provide such capabilities to non-bioinformatics experts.

>> Reviewer #4 - comment 1:
One issue that could be addressed is the use of the word "Fusion." Does this simply mean image registration between two images or is there an applied analysis where one gains information from another modality through data analysis like Nat Methods. 2015 Apr; 12(4): 366-372.

>> Authors:
We thank the reviewer for pointing this out. We have replaced "Fusion" by (image) registration. We also have done work on image fusion, but this is not yet part of the current version of M²aia and therefore not described in the paper.

>> Reviewer #4 - comment 2:
Can the authors comment on the output imzML and mention interpolation after registration? It is only briefly mentioned, and their registration methods, whether linear or non-linear will result in interpolation. It is possible the resulting "pseudo-spectra" generated after registration are no longer valid, or should at least be considered carefully, especially with regards to non-linear deformations that could potentially create large subsets of new pixels from interpolation.

>> Authors:
A discussion of this issue has been added to the Discussion section on page 10 (second last paragraph):

"With rare exceptions, transforming an image to another coordinate system requires interpolation of image data. If interpolation is applied to spectral data, the interpolated spectra must be interpreted with caution. To avoid possible misinterpretation of interpolated spectra, M2aia currently calculates only interpolated ion images and allows to store the transformation parameters for use together with the unmodified MSI data. To avoid interpolation of spectra in a multi-modal registration task with MSI and non-MSI data, the MSI image domain should be used as the fixed image domain."

>> Reviewer #4 - comment 3:
There is an important missing discussion around multi-modal MSI and microscopy data in this manuscript that could make it more powerful. This is by far the most common multi-modal MSI experiment and interactive visualization is not straightforward compared to strictly MSI datasets or even MSI-MSI multimodal data as shown in the manuscript. The authors are advantaged in their choice of toolkit as MSI data, while containing many spectral bands and typically stored as spectra rather than images, are similar to the medical images (MRI/ CT, etc.) in the spatial domain in terms of size (i.e., a few hundreds or thousands of pixels). However, the natural corollary to MSI in optical space is the whole slide image, which presents challenges in visualization (i.e., pyramidal, tiled storage that allow memory-efficient reading and fast rendering) that the authors have not demonstrated in their manuscript. It seems to exist based on a look through the code-base on GitHub and could be worth highlighting here in supplemental.

>> Authors:
We thank the reviewer for mentioning this important point. Interactive visualization of MSI data registered to Non-MSI data was not part of the 2021.02 release of M^{2}aia which is described in the manuscript. We highlight in the discussion that MSI-to-Non-MSI registration and interaction/visualization are part of the current developments of M^{2}aia and will be available in a future release.

>> Reviewer #4 - comment 4:
As a suggestion to enable adoption of the software, the authors may want to create a simple website that describes their software and has direct links to their binaries. Many non-informatics researchers may reach the GitHub repo splash page and never know where to actually download the software as the GitHub interface may seem intimidating.

>>Authors:
Thank you for this recommendation. We are going to setup a github.io page.

Close
