# Supplementary material for: M2aia—Interactive, fast, and memory-efficient analysis of 2D and 3D multi-modal mass spectrometry imaging data
Source: Gigascience. 2021 Jul 20;10(7):giab049. doi: 10.1093/gigascience/giab049 (PMC8290197; doi:10.1093/gigascience/giab049)
Supplement: giab049_Response_to_Reviewer_Comments_Revision_1 [file giab049_response_to_reviewer_comments_revision_1.pdf]

Author's Response To Reviewer Comments

Close

Dear Editors and Reviewers,
on behalf of my co-authors I like to thank you very much for the smooth and professional review process.
Kind regards,
Jonas Cordes

Close
