# Supplementary material for: M2aia—Interactive, fast, and memory-efficient analysis of 2D and 3D multi-modal mass spectrometry imaging data
Source: Gigascience. 2021 Jul 20;10(7):giab049. doi: 10.1093/gigascience/giab049 (PMC8290197; doi:10.1093/gigascience/giab049)
Supplement: giab049_Reviewer_1_Report_Original_Submission — Chris Armit -- 3/2/2021 Reviewed [file giab049_reviewer_1_report_original_submission.pdf]

Reviewer Report

Title: MÂ²aia - Interactive, fast and memory efficient analysis of 2D and 3D multi-modal mass spectrometry imaging data

Version: Original Submission **Date:** 3/2/2021

Reviewer name: Chris Armit

Reviewer Comments to Author:

This excellent Technical Note describes M2aia (MSI applications for interactive analysis in MITK), a software tool enabling interactive signal processing and visualisation of Mass spectrometry imaging (MSI) datasets. As the authors explain, M2aia extends the well used Medical Imaging and Interaction Toolkit (MITK) (Nolden et al., Journal of Computer Assisted Radiology and Surgery. 2013 Jul.). Key features of the M2aia toolkit include image segmentation, dimensionality reduction, and elastix-based image registration. Importantly, M2aia supports read/write access to the open standard imaging format mz Markup Language (imzML) that is used for MSI data.
The authors highlight the use of M2aia through two use cases. The first use case explored N-linked glycan m/z candidate detection in formalin-fixed paraffin-embedded (FFPE) murine kidney tissue. The latter 3D reference dataset has previously been published in GigaScience (Oetjen et al., GigaScience, Volume 4, Issue 1, December 2015, s13742-015-0059-4), and the authors used this reference dataset to showcase dimensionality reduction and R-based processing of MSI data. The second use case was a study of multi-modal 3D image reconstruction in 10 consecutive brain slices, and this and was used to determine the spatial distribution of brain lipids and brain peptides in an APP NL-G-F mouse model. Importantly, the supporting APP NL-G-F mouse brain lipid and protein MALDI MSI data have been submitted to the GigaScience DataBase (GigaDB), as have the intermediate results of the N-glycan MALDI mouse kidney use case study.
The M2aia software tool is made publicly available on GitHub (https://github.com/jtfcordes/m2aia) where it has been ascribed an OSI-approved BSD-3-Clause License.
I recommend this manuscript for publication in GigaScience.

Level of Interest

Please indicate how interesting you found the manuscript: Choose an item.

Quality of Written English

Please indicate the quality of language in the manuscript: Choose an item.

Declaration of Competing Interests

Please complete a declaration of competing interests, considering the following questions:

- Have you in the past five years received reimbursements, fees, funding, or salary from an organisation that may in any way gain or lose financially from the publication of this manuscript, either now or in the future?
- Do you hold any stocks or shares in an organisation that may in any way gain or lose financially from the publication of this manuscript, either now or in the future?
- Do you hold or are you currently applying for any patents relating to the content of the manuscript?
- Have you received reimbursements, fees, funding, or salary from an organization that holds or has applied for patents relating to the content of the manuscript?
- Do you have any other financial competing interests?
- Do you have any non-financial competing interests in relation to this paper?

If you can answer no to all of the above, write 'I declare that I have no competing interests' below. If your reply is yes to any, please give details below.

I declare that I have no competing interests.

I agree to the open peer review policy of the journal. I understand that my name will be included on my report to the authors and, if the manuscript is accepted for publication, my named report including any attachments I upload will be posted on the website along with the authors' responses. I agree for my report to be made available under an Open Access Creative Commons CC-BY license (http://creativecommons.org/licenses/by/4.0/). I understand that any comments which I do not wish to be included in my named report can be included as confidential comments to the editors, which will not be published.

Choose an item.

To further support our reviewers, we have joined with Publons, where you can gain additional credit to further highlight your hard work (see: https://publons.com/journal/530/gigascience). On publication of this paper, your review will be automatically added to Publons, you can then choose whether or not to claim your Publons credit. I understand this statement.

Yes Choose an item.
