# Supplementary material for: M2aia—Interactive, fast, and memory-efficient analysis of 2D and 3D multi-modal mass spectrometry imaging data
Source: Gigascience. 2021 Jul 20;10(7):giab049. doi: 10.1093/gigascience/giab049 (PMC8290197; doi:10.1093/gigascience/giab049)
Supplement: giab049_Reviewer_2_Report_Original_Submission — Liam McDonnell -- 3/9/2021 Reviewed [file giab049_reviewer_2_report_original_submission.pdf]

Reviewer Report

Title: MÂ²aia - Interactive, fast and memory efficient analysis of 2D and 3D multi-modal mass spectrometry imaging data

Version: Original Submission **Date:** 3/9/2021

Reviewer name: Liam McDonnell

Reviewer Comments to Author:

The M2aia manuscript describes a set of routines that enable fast and memory efficient analysis of 2D and 3D multimodal mass spectrometry imaging datasets. The routines consist of data input, routine mass spectral processing of [time-of-flight-based] MALDI mass spectrometry MSI data, coregistration with histology or between MSI datasets, generation and visualisation of 3D MSI datasets, and basic dimensionality reduction methods (PCA and tSNE). From the perspective of MSI itself few of the routines are innovative in their own right, for instance the data reduction methods described here parallel those this reviewer published previously [J Am Soc Mass Spectrom. 2010 21:1969-78] and which have been adopted and refined by other groups since. Similarly the registration methods are also similar to those reported previously and which are correctly cited in the current manuscript. However many of those routines have remained academic exercises that have, much to this reviewer's chagrin, not been adequately disseminated to the wider community. The principal advantage of the work is that it provides powerful open source methods that the MSI community can adopt, exploit and improve further. So while it may be lamented that the routines seem overly focused on time-of-flight based MALDI MSI this reviewer would expect them to work very well for FTICR and Orbitrap data; similarly it may be correct that the choices for mass spectral processing or data reduction are rather limited in scope, and do not represent all of the tools that have been previously used, however that would be unfair. The authors have made an open source set of routines that enable multimodal 2D and 3D MSI, and which can be supplemented with additional routines/tools. It is a commendable effort that this reviewer is more than willing to recommend is published in its current status.

Level of Interest

Please indicate how interesting you found the manuscript: Choose an item.

Quality of Written English

Please indicate the quality of language in the manuscript: Choose an item.

Declaration of Competing Interests

Please complete a declaration of competing interests, considering the following questions:

- Have you in the past five years received reimbursements, fees, funding, or salary from an organisation that may in any way gain or lose financially from the publication of this manuscript, either now or in the future?
- Do you hold any stocks or shares in an organisation that may in any way gain or lose financially from the publication of this manuscript, either now or in the future?
- Do you hold or are you currently applying for any patents relating to the content of the manuscript?
- Have you received reimbursements, fees, funding, or salary from an organization that holds or has applied for patents relating to the content of the manuscript?
- Do you have any other financial competing interests?
- Do you have any non-financial competing interests in relation to this paper?

If you can answer no to all of the above, write 'I declare that I have no competing interests' below. If your reply is yes to any, please give details below.

I declare that I have no competing interests

I agree to the open peer review policy of the journal. I understand that my name will be included on my report to the authors and, if the manuscript is accepted for publication, my named report including any attachments I upload will be posted on the website along with the authors' responses. I agree for my report to be made available under an Open Access Creative Commons CC-BY license (http://creativecommons.org/licenses/by/4.0/). I understand that any comments which I do not wish to be included in my named report can be included as confidential comments to the editors, which will not be published.

Choose an item.

To further support our reviewers, we have joined with Publons, where you can gain additional credit to further highlight your hard work (see: https://publons.com/journal/530/gigascience). On publication of this paper, your review will be automatically added to Publons, you can then choose whether or not to claim your Publons credit. I understand this statement.

Yes Choose an item.
