# Supplementary material for: M2aia—Interactive, fast, and memory-efficient analysis of 2D and 3D multi-modal mass spectrometry imaging data
Source: Gigascience. 2021 Jul 20;10(7):giab049. doi: 10.1093/gigascience/giab049 (PMC8290197; doi:10.1093/gigascience/giab049)
Supplement: giab049_Reviewer_3_Report_Original_Submission — Nathalie Agar -- 3/16/2021 Reviewed [file giab049_reviewer_3_report_original_submission.pdf]

Reviewer Report

Title: MÂ²aia - Interactive, fast and memory efficient analysis of 2D and 3D multi-modal mass spectrometry imaging data

Version: Original Submission **Date:** 3/16/2021

Reviewer name: Nathalie Agar

Reviewer Comments to Author:

The authors present an interesting software tool for interactive visualization and analysis of mass spectrometry imaging data and its integration with other imaging modalities. The paper is well presented and the authors' generosity to make it publicly available will be highly appreciated by the community. I very much appreciate the development of this tool, and below are a few suggestions that would be great to address:

1- The authors mentioned "memory efficient" analysis, but it was not clear how they implement the memory efficient algorithm. Figure 2 would need some clarification. The results show data from two case studies with relatively smaller file size (data size of one case was reported 4.9 and 2.8 GB but the file size of the other case was not reported, please add). While in Table 1, the authors referred to much larger size data, how did they analyze 44.2GB of 3D mouse kidney data with only 235.1MB RAM usage? How is peak picking efficiently performed uploading full data? Please clarify this interesting point, especially since you highlight this as a problem in the field with continuous increase in data size.

2- In the discussion, it would be great to discuss how you would envision the future development of this tool in light of analyzing big MSI data? For instance, if I have an imzML file of 100 GB or more, can I analyze it using the current version of this tool (how much memory would be needed?) or are you going to further extend it up for more scalable level?

3- Image registration: there have been great efforts already done to automate the deformable registration between mass spectrometry imaging and other modalities. The authors covered key papers in the bibliography, but it would be valuable to shed more light either in the introduction or discussion to highlight the challenges, how the published methods addressed the challenges and how your tool benefited from it (if it did), and what are the existing challenges that still need to be addressed in this active research area.

Level of Interest

Please indicate how interesting you found the manuscript: Choose an item.

Quality of Written English

Please indicate the quality of language in the manuscript: Choose an item.

Declaration of Competing Interests

Please complete a declaration of competing interests, considering the following questions:

- Have you in the past five years received reimbursements, fees, funding, or salary from an organisation that may in any way gain or lose financially from the publication of this manuscript, either now or in the future?
- Do you hold any stocks or shares in an organisation that may in any way gain or lose financially from the publication of this manuscript, either now or in the future?
- Do you hold or are you currently applying for any patents relating to the content of the manuscript?
- Have you received reimbursements, fees, funding, or salary from an organization that holds or has applied for patents relating to the content of the manuscript?
- Do you have any other financial competing interests?
- Do you have any non-financial competing interests in relation to this paper?

If you can answer no to all of the above, write 'I declare that I have no competing interests' below. If your reply is yes to any, please give details below.

I declare that I have no competing interests

I agree to the open peer review policy of the journal. I understand that my name will be included on my report to the authors and, if the manuscript is accepted for publication, my named report including any attachments I upload will be posted on the website along with the authors' responses. I agree for my report to be made available under an Open Access Creative Commons CC-BY license (http://creativecommons.org/licenses/by/4.0/). I understand that any comments which I do not wish to be included in my named report can be included as confidential comments to the editors, which will not be published.

Choose an item.

To further support our reviewers, we have joined with Publons, where you can gain additional credit to further highlight your hard work (see: https://publons.com/journal/530/gigascience). On publication of this paper, your review will be automatically added to Publons, you can then choose whether or not to claim your Publons credit. I understand this statement.

Yes Choose an item.
