# Supplementary material for: M2aia—Interactive, fast, and memory-efficient analysis of 2D and 3D multi-modal mass spectrometry imaging data
Source: Gigascience. 2021 Jul 20;10(7):giab049. doi: 10.1093/gigascience/giab049 (PMC8290197; doi:10.1093/gigascience/giab049)
Supplement: giab049_Reviewer_4_Report_Original_Submission — Nathan Heath Patterson -- 3/22/2021 Reviewed [file giab049_reviewer_4_report_original_submission.pdf]

Reviewer Report

Title: MÂ^{2}aia - Interactive, fast and memory efficient analysis of 2D and 3D multi-modal mass spectrometry imaging data

Version: Original Submission **Date:** 3/22/2021

Reviewer name: Nathan Heath Patterson

Reviewer Comments to Author:

The authors describe their software for MSI visualization and interactive analysis that is built on an existing image visualization and analysis framework, integrating the strength and development of the medical imaging community with MSI data.
Overall, this appears to be an excellent work and responds to the need and desires of the MSI community. While there have been multiple publications on 3D MSI, an open-source tool with a GUI for human-in-the-loop preprocessing and setup has yet to emerge and the described software can meet that need and provide such capabilities to non-bioinformatics experts.
One issue that could be addressed is the use of the word "Fusion." Does this simply mean image registration between two images or is there an applied analysis where one gains information from another modality through data analysis like Nat Methods. 2015 Apr; 12(4): 366-372.
Can the authors comment on the output imzML and mention interpolation after registration? It is only briefly mentioned, and their registration methods, whether linear or non-linear will result in interpolation. It is possible the resulting "pseudo-spectra" generated after registration are no longer valid, or should at least be considered carefully, especially with regards to non-linear deformations that could potentially create large subsets of new pixels from interpolation.
There is an important missing discussion around multi-modal MSI and microscopy data in this manuscript that could make it more powerful. This is by far the most common multi-modal MSI experiment and interactive visualization is not straightforward compared to strictly MSI datasets or even MSI-MSI multimodal data as shown in the manuscript. The authors are advantaged in their choice of toolkit as MSI data, while containing many spectral bands and typically stored as spectra rather than images, are similar to the medical images (MRI/ CT, etc.) in the spatial domain in terms of size (i.e., a few hundreds or thousands of pixels). However, the natural corollary to MSI in optical space is the whole slide image, which presents challenges in visualization (i.e., pyramidal, tiled storage that allow memory-efficient reading and fast rendering) that the authors have not demonstrated in their manuscript. It seems to exist based on a look through the code-base on GitHub and could be worth highlighting here in supplemental.
As a suggestion to enable adoption of the software, the authors may want to create a simple website that describes their software and has direct links to their binaries. Many non-informatics researchers may reach the GitHub repo splash page and never know where to actually download the software as the GitHub interface may seem intimidating.

Level of Interest

Please indicate how interesting you found the manuscript: Choose an item.

Quality of Written English

Please indicate the quality of language in the manuscript: Choose an item.

Declaration of Competing Interests

Please complete a declaration of competing interests, considering the following questions:

- Have you in the past five years received reimbursements, fees, funding, or salary from an organisation that may in any way gain or lose financially from the publication of this manuscript, either now or in the future?
- Do you hold any stocks or shares in an organisation that may in any way gain or lose financially from the publication of this manuscript, either now or in the future?
- Do you hold or are you currently applying for any patents relating to the content of the manuscript?
- Have you received reimbursements, fees, funding, or salary from an organization that holds or has applied for patents relating to the content of the manuscript?
- Do you have any other financial competing interests?
- Do you have any non-financial competing interests in relation to this paper?

If you can answer no to all of the above, write 'I declare that I have no competing interests' below. If your reply is yes to any, please give details below.

I declare that I have no competing interests.

I agree to the open peer review policy of the journal. I understand that my name will be included on my report to the authors and, if the manuscript is accepted for publication, my named report including any attachments I upload will be posted on the website along with the authors' responses. I agree for my report to be made available under an Open Access Creative Commons CC-BY license (http://creativecommons.org/licenses/by/4.0/). I understand that any comments which I do not wish to be included in my named report can be included as confidential comments to the editors, which will not be published.

Choose an item.

To further support our reviewers, we have joined with Publons, where you can gain additional credit to further highlight your hard work (see: https://publons.com/journal/530/gigascience). On publication of this paper, your review will be automatically added to Publons, you can then choose whether or not to claim your Publons credit. I understand this statement.

Yes Choose an item.
